# ARTS Confers Chemoresistance of Breast Cancer by Inducing Apoptosis-Dependent Autophagy via Livin–MDM2–p53 Pathway

**DOI:** 10.34133/research.1086

**Published:** 2026-01-15

**Authors:** Hao Wang, Qianying Guo, Yuting Shen, Keshuo Ding, Yinfeng Chen, Xiaonan Wang, Xing Huang, Zhengsheng Wu

**Affiliations:** ^1^ Department of Pathology, School of Basic Medicine, Anhui Medical University, Hefei, Anhui, China.; ^2^ Department of Pathology, The First Affiliated Hospital of Anhui Medical University, Hefei, Anhui, China.; ^3^ Zhejiang Provincial Key Laboratory of Pancreatic Disease, The First Affiliated Hospital, School of Medicine, Zhejiang University, Hangzhou, Zhejiang, China.; ^4^ Laboratory of Pathogenic Microbiology and Immunology, School of Basic Medicine, Anhui Medical University, Hefei, Anhui, China.

## Abstract

Apoptosis and autophagy are fundamental pathophysiological programs governing cell fate decisions under stress, particularly during anticancer therapy. However, the interplay between apoptosis and autophagy in cancer chemoresistance remains incompletely understood. Here, we identify the apoptosis-related protein in the transforming growth factor-β signaling pathway (ARTS) as a key molecular transferring apoptotic signal to autophagic machinery to promote cell survival and chemoresistance. ARTS was highly expressed in chemoresistant breast cancer tissues and was associated with poor patient prognosis. ARTS conferred resistance to doxorubicin and docetaxel by inducing protective autophagy in vitro and in vivo cancer models. Mechanistically, upon proapoptotic signaling triggered by chemotherapeutic agents, ARTS translocated from the mitochondrial intermembrane space into the cytosol, where it induced autophagy through triggering seven in absentia homolog 1-mediated degradation of Livin and subsequent engagement of the mouse double minute 2 homolog (MDM2)–p53 axis, thereby promoting cancer cell survival. Pharmacologic inhibition of caspases or autophagic flux attenuated ARTS-mediated chemoresistance. Overall, this study delineates an apoptosis-dependent ARTS–Livin–MDM2–p53 pathway that drives autophagy and confers chemoresistance in breast cancer.

## Introduction

Chemotherapy has long been a cornerstone of cancer treatment, targeting rapidly dividing cancer cells in many tumor types [1]. It has led to improved survival rates, marked tumor reduction, and, in some instances, complete remission [2]. However, cancer cells can develop intrinsic or acquired resistance through various mechanisms, creating a major obstacle to effective treatment and substantially limiting its efficacy [3].

Many chemotherapeutic agents eliminate tumor cells by inducing apoptosis [4]. Apoptosis is a genetically encoded cell death program executed by caspases, which orchestrate signaling cascades that rapidly dismantle cellular structures and organelles, thereby maintaining organismal homeostasis [5]. For example, apoptosis removes damaged or mutated cells, preventing malignant transformation and cancer initiation [6]. During development, physiological apoptosis shapes organ structures and limb formation (morphogenesis) [7]. Apoptosis also contributes to immune tolerance and the regulation of immune responses [8]. These multifaceted roles underscore its essentiality for organismal health and survival [5]. Paradoxically, apoptosis-related signaling can also contribute to treatment failure in clinical settings [9]. For instance, after radiotherapy, apoptotic tumor cells may release mitogenic factors that drive tumor repopulation (apoptosis-induced proliferation) [10]. Thus, the net contribution of apoptosis to therapeutic resistance remains context-dependent and incompletely understood, highlighting the need to delineate when and how apoptotic signaling constrains or promotes chemoresistance.

Apoptosis-related protein in the transforming growth factor-β (TGF-β) signaling pathway (ARTS), also known as Septin-4 isoform 2, is a mitochondria-associated, proapoptotic splice isoform encoded by *SEPTIN4* and was first identified for its role in TGF-β-induced apoptosis in the rat prostate epithelial cell line NRP-154 [11]. TGF-β regulates diverse cellular processes, including control of proliferation and apoptosis and maintenance of tissue homeostasis [12]. ARTS functions downstream of TGF-β signaling, particularly in the regulation of apoptosis [13]. Upon proapoptotic signaling and mitochondrial outer membrane permeabilization (MOMP), ARTS is released from mitochondria into the cytosol, where it binds the baculoviral IAP repeat 1 (BIR1) and BIR3 domains of X-linked inhibitor of apoptosis protein (XIAP), thereby antagonizing XIAP [14]. ARTS also promotes seven in absentia homolog 1 (SIAH1)-dependent ubiquitination and degradation of XIAP [15]. Importantly, ARTS translocation precedes the release of cytochrome c and SMAC/DIABLO and marks an early apoptotic event [16]. Moreover, ARTS can interact directly with p53, facilitate its mitochondrial translocation, and enhance its association with B cell lymphoma-extra large (BCL-xL), thereby activating the mitochondrial apoptotic pathway [17]. The physiological roles of ARTS have been explored in genetic mouse models [18]: Mice lacking ARTS display markedly faster wound healing and hair follicle regeneration, owing to protection of hair follicle stem cells from apoptosis [13]; cells of the intestinal stem cell niche express high levels of ARTS, and its deletion protects intestinal stem and progenitor cells from apoptosis [19]. Notably, limited (minority) MOMP has been reported to stimulate autophagy, extend lifespan, and promote cellular transformation and tumorigenesis [20,21]. However, it remains incompletely understood how mitochondrial apoptosis and autophagy intersect to shape cancer therapeutic resistance and whether ARTS participates in this cross-talk.

This study, in the context of breast cancer, reveals that apoptosis-triggered ARTS-mediated autophagy enables tumor cells to evade chemotherapy-induced cell death through SIAH1–Livin–MDM2–p53 axis, highlighting ARTS as a potential therapeutic target for overcoming chemoresistance.

## Results

### ARTS, a mitochondrial apoptotic protein, is a potential promoter of breast cancer chemoresistance

**Fig. 1.**
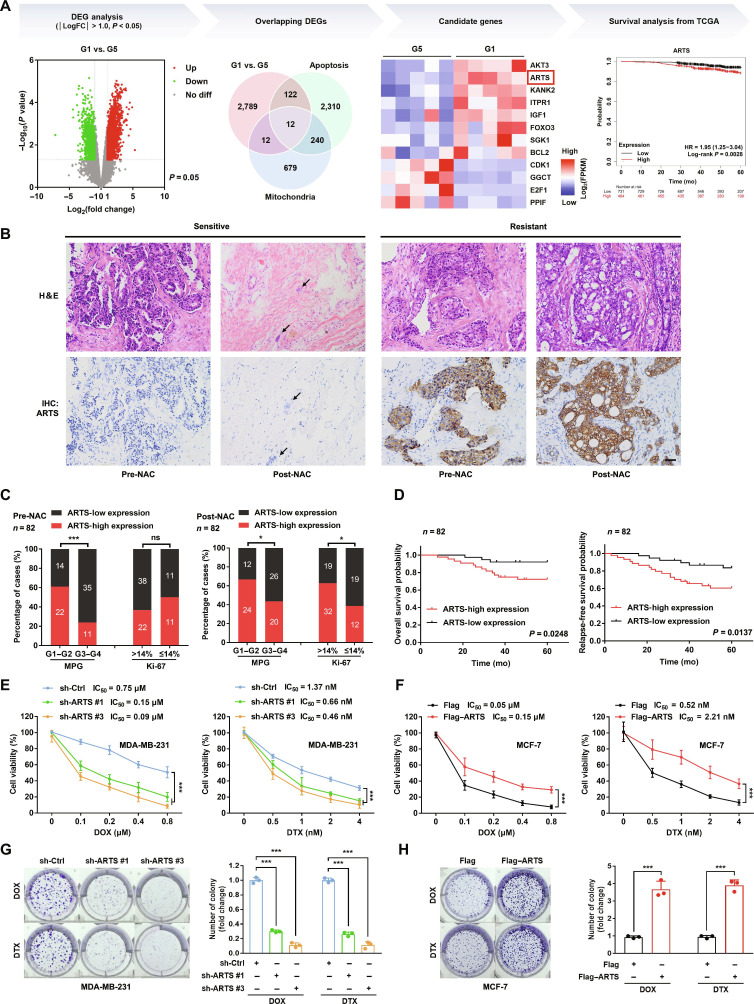
ARTS is predominantly expressed in resistant breast cancer tissues and promotes chemoresistance in breast cancer cells. (A) Screening workflow for chemoresistance-related genes in breast cancer. DEGs from GSE288073 are shown in a volcano plot, followed by overlap with apoptosis-related and mitochondria-related gene sets, yielding 12 DEGs; ARTS was prioritized and evaluated by Kaplan–Meier survival analysis for postchemotherapy prognosis. FPKM, fragments per kilobase of transcript per million mapped reads. (B) Representative hematoxylin and eosin (H&E) and IHC images of ARTS in paired pre- and post-NAC samples from NAC-sensitive and NAC-resistant patients. The black arrow indicates a residual small cluster of tumor cells. Scale bar, 50 μm. (C) Association of ARTS protein with MPG score and Ki-67 in pre- and post-NAC samples. (D) Kaplan–Meier analyses of OS and RFS stratified by ARTS protein (low versus high). (E and F) MTT and (G and H) colony formation assays in sh-Ctrl versus sh-ARTS MDA-MB-231 and Flag versus Flag–ARTS MCF-7 cells under the indicated treatments. *P < 0.05; ***P < 0.001. ns, not significant.

To investigate the role of apoptosis in chemoresistance, we evaluated paired breast cancer tissues collected before or after neoadjuvant chemotherapy (pre- or post-NAC) using the Miller–Payne grading (MPG) system and performed RNA sequencing (RNA-seq) on pre-NAC core needle biopsy samples from 5 G5 and 5 G1 patients (Fig. 1A). The RNA-seq data have been deposited in National Center for Biotechnology Information Gene Expression Omnibus (GEO) (accession GSE288073). From 3 datasets (GSE288073, apoptosis-related gene set, and mitochondria-related gene set), we identified 12 overlapping differentially expressed genes (DEGs) using the thresholds log_2_ fold change (|log_2_FC|) > 1.0 and *P* < 0.05. We selected ARTS for further study because it ranked highly in our screen and showed a significant association with breast cancer prognosis in The Cancer Genome Atlas (TCGA) cohort by Kaplan–Meier analysis.

We next examined ARTS expression in paired tissues by immunohistochemistry (IHC). Compared with samples from NAC-responsive patients, NAC-resistant tumors exhibited higher ARTS protein levels (Fig. 1B and C and Fig. S1A). Specifically, ARTS-high samples were more frequent both pre- and post-NAC in patients with low MPG scores, a clinical indicator of poor pathological response. Consistently, higher ARTS levels were significantly associated with a higher proliferative index as defined by Ki-67 in post-NAC tumor samples. By contrast, ARTS expression showed no significant association with patient age, lymph node status, tumor stage, or estrogen receptor (ER)/progesterone receptor (PR)/human epidermal growth factor receptor 2 (HER2) status (Table S1). In line with these observations, ARTS expression was up-regulated in doxorubicin (DOX)-resistant MCF-7 (MCF-7/DOX) cells compared with parental MCF-7 cells (Fig. S1B and C). A combined analysis of 3 neoadjuvant cohorts (GSE20194/25055/194040) showed that ARTS mRNA was significantly higher in patients with residual disease (*n* = 226) than in those achieving pathologic complete response (*n* = 126) (Fig. S1D). Kaplan–Meier analysis in our breast cancer cohort (*n* = 82) further revealed a significant correlation between ARTS protein and overall survival (OS) as well as recurrence-free survival (RFS) (Fig. 1D).

We profiled endogenous ARTS across a panel of breast cancer cell lines by immunoblot (Fig. S1E) and tested its functional role using ARTS-knockdown MDA-MB-231 cells (Fig. S1F) and ARTS-overexpressing MCF-7 cells (Fig. S1G) treated with DOX or docetaxel (DTX). ARTS knockdown reduced, whereas ARTS overexpression increased, resistance to DOX/DTX as measured by cell viability (Fig. 1E and F) and colony formation (Fig. 1G and H); no significant differences were observed in the absence of drug (Fig. S1H to K). Collectively, these results indicate that ARTS is enriched in chemoresistant breast cancer and suggest that ARTS modulates cellular sensitivity to chemotherapeutic agents.

### Chemotherapy-induced ARTS relocalization promotes chemoresistance

ARTS has been reported to relocalize from mitochondria into the cytosol at the onset of apoptosis in a MOMP-dependent manner [14]. Given the apoptosis-inducing nature of chemotherapy, we first mapped ARTS localization by confocal immunofluorescence, costaining ARTS with the mitochondrial marker translocase of outer mitochondrial membrane 20 (TOM20) in cells exposed to chemotherapeutic agents across a range of doses (20% inhibitory concentration [IC_20_] to IC_80_) and time points (2, 4, 8, 16, and 24 h), alongside untreated controls. Under untreated conditions, ARTS showed robust mitochondrial residency, as evidenced by strong colocalization with TOM20 and high Pearson’s correlation coefficient (PCC) values (Fig. S2A). Exposure to DOX or DTX markedly reduced ARTS–TOM20 colocalization, indicating mitochondrial-to-cytosolic relocalization under chemotherapeutic stress. Across dose–response tests, IC_50_ produced clearer and more consistent PCC changes than IC_20_, while mirroring the direction of IC_80_ at a lower concentration; therefore, IC_50_ was used in subsequent experiments. A time-course analysis revealed a progressive PCC decline that paralleled classical apoptotic readouts (cleaved poly[adenosine-diphosphate-ribose] polymerase and cleaved caspase-3) and loss of mitochondrial membrane potential (JC-1, a mitochondrial membrane potential [ΔΨm]–sensitive dye), supporting a temporal linkage between apoptosis onset and ARTS relocalization (Fig. S2B and C).

**Fig. 2.**
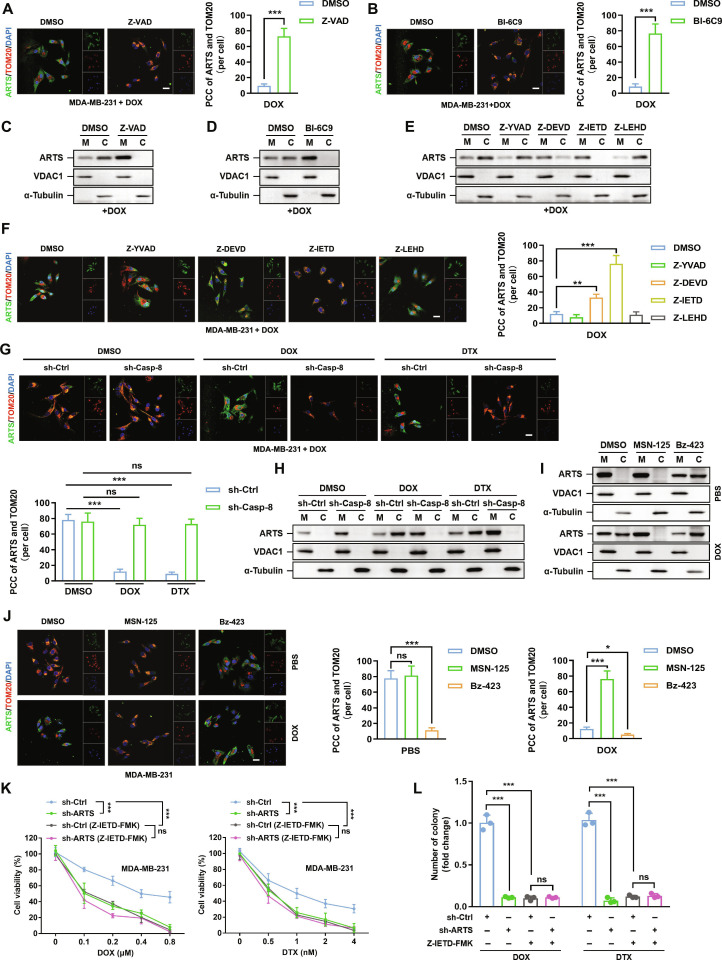
Chemotherapy-induced ARTS relocalization promotes chemoresistance. (A and B) Confocal imaging of ARTS (green) and TOM20 (red) in MDA-MB-231 cells exposed to DOX with DMSO versus Z-VAD-FMK (A) or DMSO versus BI-6C9 (B). Right: Single-cell PCC for ARTS–TOM20. DAPI, nuclei. Scale bars, 20 μm. (C to E) Cytosolic (C)/mitochondrial (M) fractionation in MDA-MB-231 cells after DOX with Z-VAD-FMK (C), BI-6C9 (D), or selective caspase inhibitors Z-YVAD-FMK (a caspase-1 inhibitor), Z-DEVD-FMK (a caspase-3/7 inhibitor), Z-IETD-FMK, and Z-LEHD-FMK (a caspase-9 inhibitor) (E). Voltage-dependent anion channel 1 (VDAC1) and α-tubulin mark mitochondrial and cytosolic fractions, respectively. (F) Immunofluorescence (IF) as in (A) and (B) showing the caspase inhibitor panel. Right: PCC quantification. Scale bar, 20 μm. (G and H) Genetic inhibition of caspase-8 in MDA-MB-231 cells: sh-Ctrl or sh-Casp8 cells treated with DMSO, DOX, or DTX. Left: ARTS–TOM20 immunofluorescence with PCC. Right: Fractionation blots. Scale bar, 20 μm. (I and J) Modulation of Bax/Bak-dependent MOMP in MDA-MB-231 cells. Cells treated with MSN-125 (Bax/Bak inhibitor) or Bz-423 (activator) under PBS or DOX. (I) Fractionation; (J) immunofluorescence with PCC for PBS and DOX. Scale bar, 20 μm. (K) Cell viability of sh-Ctrl and sh-ARTS MDA-MB-231 cells treated with DOX or DTX (±Z-IETD-FMK). (L) Colony formation under the same conditions as (K). *P < 0.05; **P < 0.01; ***P < 0.001. ns, not significant.

Broad caspase blockade (Z-VAD-FMK, a pan-caspase inhibitor) or inhibition of the BH3-interacting domain death agonist (BID)/truncated BID (tBID) axis (BI-6C9) restored ARTS–TOM20 colocalization and prevented cytosolic accumulation under DOX/DTX, with concordant subcellular fractionation evidence showing preserved mitochondrial ARTS and diminished cytosolic ARTS (Fig. 2A to D and Fig. S3A to D). Among selective caspase inhibitors, Z-IETD-FMK (a caspase-8 inhibitor) exerted the strongest effect, implicating caspase-8 as the principal driver. Consistently, short hairpin RNA (shRNA) targeting caspase-8 (sh-Casp-8) reproduced these effects in both immunofluorescence and fractionation assays (Fig. 2E to H and Fig. S3E and F), supporting a caspase-8-dependent initiation.

To confirm the involvement of MOMP, we cotreated cells with chemotherapy and either the Bcl-2–associated X protein (Bax)/Bcl-2 antagonist/killer 1 (Bak) inhibitor MSN-125 or the activator Bz-423. Both Bz-423 and chemotherapeutic drugs induced ARTS relocalization from mitochondria into the cytosol (Fig. 2I and J and Fig. S3G and H). Notably, Bz-423 further enhanced chemotherapy-induced cytosolic ARTS, whereas MSN-125 abolished this effect, confirming dependence on Bax/Bak-mediated MOMP. Importantly, this relocalization was not triggered by nonapoptotic stressors, including Earle's balanced salt solution (EBSS) starvation, hypoxia, or mechanistic target of rapamycin inhibition, indicating specificity for apoptotic signaling rather than a general stress response (Fig. S3I).

Functionally, ARTS relocalization was associated with reduced chemosensitivity. ARTS knockdown increased sensitivity to DOX/DTX and suppressed clonogenic survival, whereas ARTS overexpression produced the opposite effect. Moreover, Z-IETD-FMK or Z-VAD-FMK attenuated the differences between ARTS-altered and control cells in both short-term viability and long-term colony formation (Fig. 2K and L and Figs. S3J to L and S6A to F), indicating that the survival advantage conferred by ARTS depends on upstream apoptotic signaling. Together, these results support a model in which DOX/DTX engage a caspase-8–BID–Bax/Bak (MOMP) cascade to mobilize ARTS from mitochondria into the cytosol; cytosolic ARTS, in turn, promotes persistence under chemotherapeutic stress, thereby fostering resistance.

### Apoptosis-dependent autophagy driven by ARTS leads to chemoresistance

**Fig. 3.**
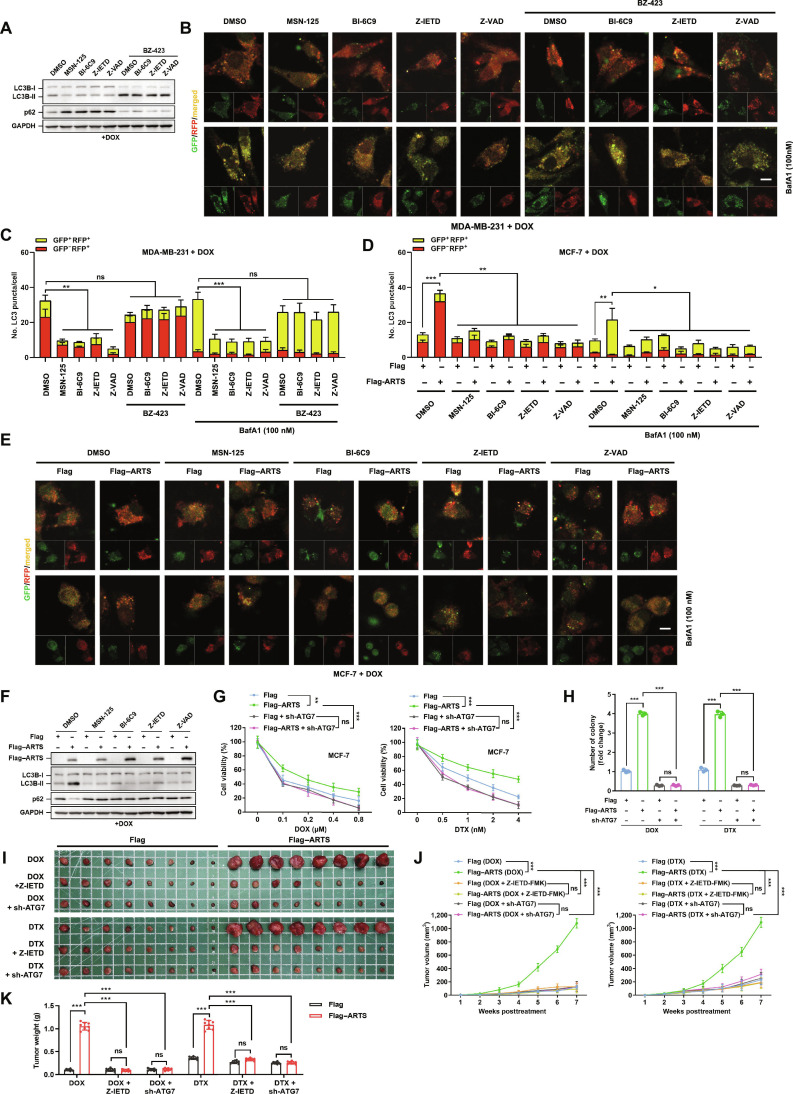
ARTS drives apoptosis-dependent autophagy and chemoresistance. (A) Immunoblot of LC3B-I/II and p62 in MDA-MB-231 cells treated with DOX plus the indicated modulators (MSN-125, BI-6C9, Z-IETD-FMK, and Z-VAD-FMK; Bz-423 shown separately). GAPDH, glyceraldehyde-3-phosphate dehydrogenase. (B) Representative mRFP–GFP–LC3 images under DOX with the same modulators ± BafA1 (100 nM, 3 h). Scale bar, 10 μm. (C) Quantification of yellow (GFP^+^RFP^+^) and red-only (RFP^+^) puncta per cell for (B) in MDA-MB-231. (D) Flux quantification in MCF-7 cells expressing Flag or Flag–ARTS with the indicated modulators ± BafA1. (E) Representative LC3 reporter images for (D). Scale bar, 10 μm. (F) Immunoblot of LC3B and p62 in Flag/Flag–ARTS MCF-7 cells under the indicated treatments. (G) MTT curves for MCF-7 Flag or Flag–ARTS ± sh-ATG7 under DOX (left) or DTX (right). (H) Corresponding colony formation. (I to K) Orthotopic in vivo model. Flag or Flag–ARTS MCF-7 cells were implanted; mice received DOX or DTX alone or combined with Z-IETD-FMK or sh-ATG7 twice weekly for 6 weeks beginning in week 2. (I) Representative tumors; (J) tumor growth curves; (K) terminal tumor weight (*n* = 8). *P < 0.05; **P < 0.01; ***P < 0.001.

Prompted by reports that minority MOMP can promote tumorigenesis by stimulating autophagy [21], we examined how chemotherapeutic agents and upstream apoptotic pathways affect autophagy in breast cancer cells. Treatment with DOX or DTX enhanced autophagic flux, as evidenced by the tandem monomeric red fluorescent protein–green fluorescent protein–LC3 (mRFP–GFP–LC3) reporter (increased puncta with or without bafilomycin A1 [BafA1]) and by concordant LC3B-II accumulation with p62 degradation on immunoblot (Fig. S4A and B). Pharmacological blockade of apoptosis or MOMP (Z-IETD-FMK, Z-VAD-FMK, BI-6C9, or MSN-125) significantly attenuated these changes (Fig. 3A to C and Fig. S4C and D), indicating that chemotherapy-induced autophagy is apoptosis/MOMP-dependent rather than a parallel, apoptosis-independent response.

Under DOX/DTX, ARTS overexpression was sufficient to increase autophagic flux (tandem reporter) and elevate LC3B-II with reciprocal p62 loss (Fig. 3D to F and Figs. S4E and F and S5A and B). These effects were abrogated by caspase inhibition or MOMP blockade, placing cytosolic ARTS downstream of the apoptotic trigger. Consistently, genetic perturbation of autophagy modulated drug response in ARTS-altered cells: autophagy related 7 (ATG7) knockdown suppressed ARTS-driven autophagy and reversed ARTS-induced drug resistance, whereas ATG7 reexpression restored flux and reinstated resistance when ARTS was depleted (Fig. 3G and H and Fig. S5C to E). The lysosomotropic inhibitor chloroquine (CQ) likewise restored DOX/DTX sensitivity across conditions, supporting a requirement for intact autophagic flux in the ARTS phenotype (Fig. S6A to F).

We corroborated these findings in vivo using an orthotopic mammary fat pad model. Tumors derived from Flag–ARTS cells exhibited diminished responses to DOX/DTX relative to vector controls, indicative of chemoresistance. Notably, combining chemotherapy with Z-IETD-FMK or sh-ATG7 significantly restrained tumor growth and reduced terminal tumor weight versus chemotherapy alone, effectively reversing ARTS-mediated resistance (Fig. 3I to K). We also observed mitochondrial-to-cytosolic relocalization of exogenous ARTS within tumors (Fig. S5F and G). Together, these data support a model in which DOX/DTX engage a caspase-8–BID–Bax/Bak (MOMP) cascade to mobilize ARTS from mitochondria into the cytosol; cytosolic ARTS then converts the apoptotic trigger into a prosurvival autophagic program, thereby undermining chemotherapy efficacy.

### ARTS regulates the ubiquitination degradation of Livin through SIAH1

**Fig. 4.**
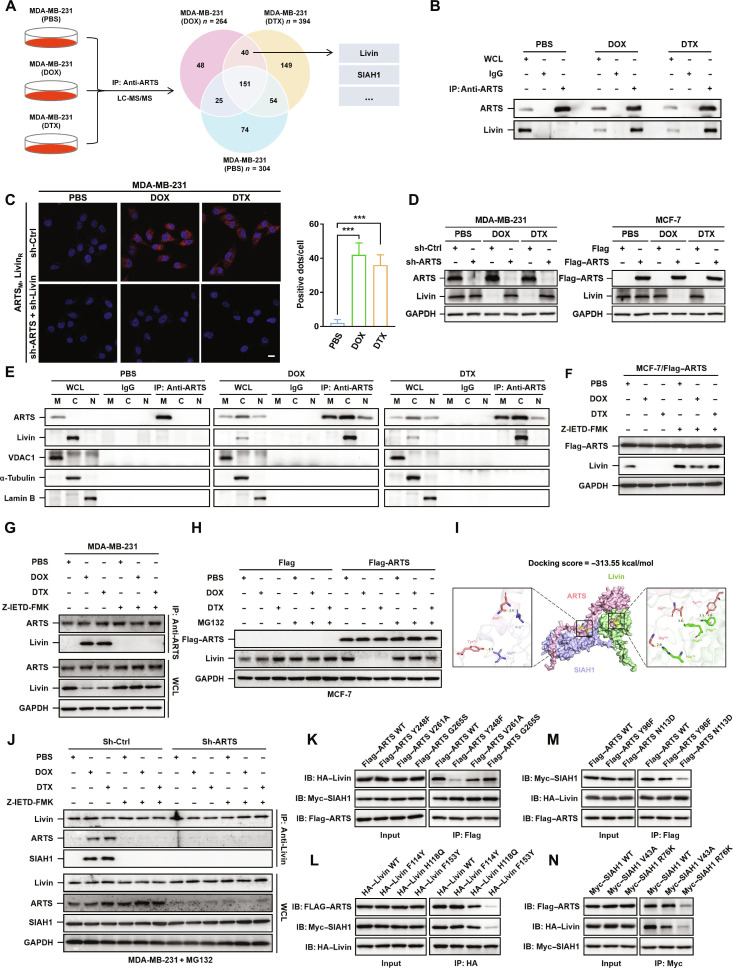
ARTS regulates the ubiquitination degradation of Livin through SIAH1. (A) Endogenous ARTS co-IP/LC-MS/MS in MDA-MB-231 cells treated with PBS, DOX, or DTX identified 40 DOX/DTX-shared interactors, including Livin and SIAH1 (Venn diagram). (B) DOX/DTX, but not PBS, induced an endogenous ARTS–Livin interaction (anti-ARTS IP). (C) Duolink PLA visualizing ARTS–Livin proximity under PBS, DOX, or DTX. Right: Positive dots per cell quantified from 30 cells. Scale bar, 20 μm. (D) Immunoblot: sh-ARTS increases, while Flag–ARTS decreases, Livin under chemotherapy. (E) Subcellular fractionation and anti-ARTS IP show that the ARTS–Livin complex forms in the cytosol upon DOX/DTX. (F and G) Z-IETD-FMK prevents DOX/DTX-induced Livin reduction (F) and blocks the endogenous ARTS–Livin interaction (G). (H) MG132 rescues Livin loss in Flag–ARTS MCF-7 cells under chemotherapy, indicating proteasome-dependent turnover. (I) Docking model of ARTS (pink), Livin (green), and SIAH1 (blue) predicts a ternary assembly (docking score, ~313.55 kcal/mol). (J) sh-Ctrl and sh-ARTS MDA-MB-231 cells were treated as indicated, subjected to IP with anti-Livin antibody, and then immunoblot was performed. (K to N) HEK293T cells were cotransfected with epitope-tagged constructs (Flag–ARTS WT or mutants, hemagglutinin [HA]–Livin WT or mutants, and Myc–SIAH1 WT or mutants). Lysates were subjected to IP with anti-Flag (K and M), anti-HA (L), or anti-Myc (N), followed by immunoblot (IB). ***P < 0.001.

To dissect the mechanism by which ARTS drives autophagy and chemoresistance, we performed coimmunoprecipitation (co-IP) of ARTS in MDA-MB-231 cells treated with phosphate-buffered saline (PBS), DOX, or DTX, followed by liquid chromatography-tandem mass spectrometry (LC-MS/MS). Proteins present in the DOX/DTX groups but absent in PBS yielded 40 candidates that met our predefined filters (Fig. 4A, Fig. S7A, and Table S2). On the basis of public database analyses and the similarity of Livin (*BIRC7*) to XIAP [14], we hypothesized that Livin might act downstream of ARTS (Fig. S7B). Indeed, ARTS–Livin interactions were detected by co-IP in DOX/DTX-treated, but not PBS-treated, MDA-MB-231 cells (Fig. 4B) and were validated by proximity ligation assay (PLA) (Fig. 4C).

We next examined Livin abundance under ARTS perturbation. In the presence of DOX/DTX, ARTS knockdown in MDA-MB-231 cells increased Livin, whereas ARTS overexpression in MCF-7 cells reduced Livin (Fig. 4D). Subcellular fractionation (nuclear/cytosolic/mitochondrial) confirmed chemotherapy-induced relocalization of ARTS from mitochondria into the cytosol (Fig. 4E), indicating that ARTS–Livin complexes form in the cytosolic fraction during DOX/DTX exposure. In MCF-7 cells, ARTS overexpression decreased Livin under DOX/DTX, an effect reversed by the caspase-8 inhibitor Z-IETD-FMK (Fig. 4F); Z-IETD-FMK likewise disrupted ARTS–Livin interactions in MDA-MB-231 cells (Fig. 4G), indicating caspase-dependent engagement.

Domain mapping in human embryonic kidney (HEK) 293T cells showed that the C-terminal 68 amino acids of ARTS mediate binding to the BIR domain of Livin, based on co-IP using Flag- or GFP-tagged wild-type (WT) and deletion mutants (Fig. S8A and B).

Treatment with the proteasome inhibitor MG132 stabilized Livin (Fig. 4H), consistent with ubiquitin-mediated degradation. Among the LC-MS/MS candidates, we focused on SIAH1 because ARTS was reported to recruit this E3 ubiquitin ligase to promote XIAP ubiquitination and degradation [15]. Molecular docking supported a model in which ARTS can simultaneously engage Livin and SIAH1, suggesting an adaptor role for ARTS within a trimeric complex (Fig. 4I). Accordingly, chemotherapy induced Livin–SIAH1 interactions in sh-Ctrl MDA-MB-231 cells, whereas sh-ARTS abrogated these interactions; Z-IETD-FMK similarly blocked Livin–SIAH1 binding in DOX/DTX-treated sh-Ctrl cells (Fig. 4J). Co-IP series using epitope-tagged ARTS, Livin, and SIAH1 (including interface-altering mutants) confirmed pairwise associations among all 3 proteins, supporting the formation of an ARTS–Livin–SIAH1 complex upon chemotherapy (Fig. 4K to N). These domain maps align with the docking predictions and provide a mechanistic basis for ARTS-guided recruitment of SIAH1 to Livin.

Ubiquitination assays further showed that DOX/DTX + MG132 markedly increased Livin ubiquitination in sh-Ctrl MDA-MB-231 cells, whereas sh-ARTS blunted this effect (Fig. S8C). ARTS overexpression in MCF-7 cells enhanced Livin ubiquitination. Consistently, SIAH1 knockdown (MDA-MB-231) reduced, while SIAH1 overexpression (MCF-7) increased, Livin ubiquitination (Fig. S8D).

Collectively, these data indicate that ARTS acts as a molecular bridge under chemotherapeutic stress, recruiting SIAH1 to Livin to drive its ubiquitin-mediated degradation.

### ARTS promotes apoptosis-dependent autophagy and chemoresistance via suppression of Livin

**Fig. 5.**
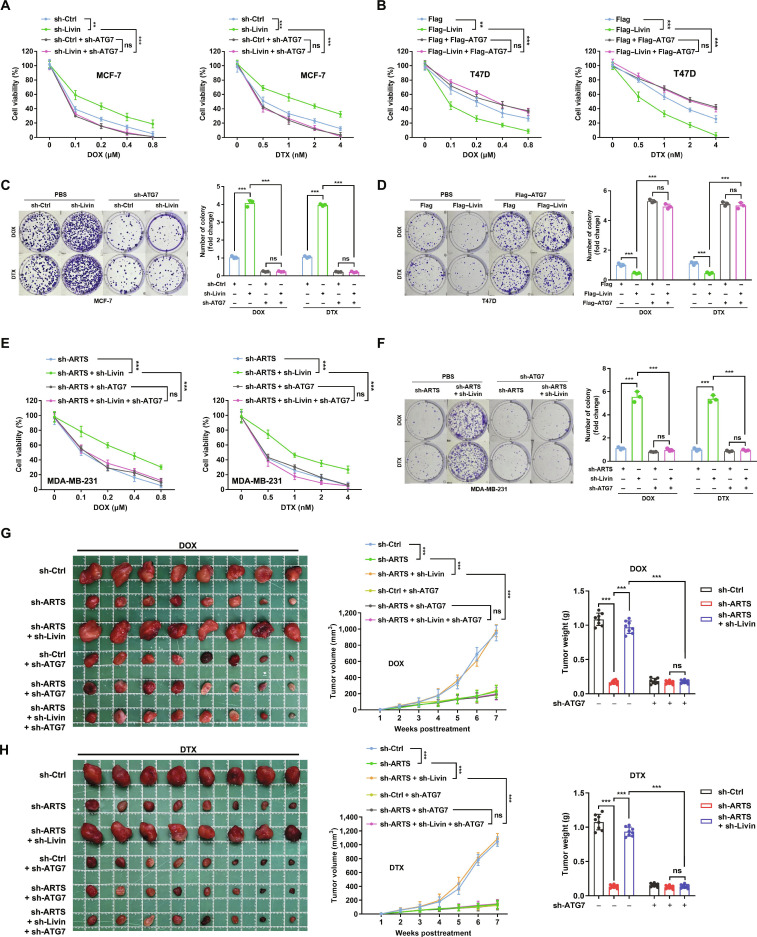
ARTS promotes apoptosis-dependent autophagy and chemoresistance via Livin. (A and B) MTT and (C and D) colony formation assays in sh-Ctrl versus sh-Livin MCF-7 cells and Flag versus Flag–Livin T47D cells under the indicated treatments. (E) MTT and (F) colony formation in sh-Ctrl versus sh-ARTS MDA-MB-231 cells cotransfected with sh-Livin or sh-ATG7 and treated as indicated. (G and H) sh-Ctrl and sh-ARTS MDA-MB-231 cells transfected with sh-Livin or sh-ATG7 were injected into the second pair of mammary fat pads of nude mice. DOX or DTX was administered twice weekly for 6 weeks starting in week 2 (when tumors reached ~50 mm^3^). Tumor growth curves, representative tumor images, and terminal weights are shown (*n* = 8). **P < 0.01; ***P < 0.001.

We next examined how altering Livin affects cell behavior and chemoresistance. Cell viability and colony formation assays were performed under DOX or DTX treatment, with or without sh-ATG7. Livin knockdown significantly increased chemoresistance, and this effect was abrogated by ATG7 knockdown (Fig. 5A and C). Conversely, Livin overexpression decreased chemoresistance, whereas ATG7 coexpression restored resistance (Fig. 5B and D). Under all conditions, cotreatment with CQ resensitized cells to chemotherapy (Fig. S9A to I).

We then interrogated the ARTS–Livin–autophagy axis in drug response. ARTS depletion significantly reduced chemoresistance in MDA-MB-231 cells, and Livin knockdown reversed this sensitization in both viability and clonogenic assays (Fig. 5E and F). The increased resistance driven by the ARTS–Livin axis was suppressed by ATG7 knockdown, and CQ cotreatment restored chemosensitivity across groups (Fig. S10A and B).

The in vivo relevance of this pathway was evaluated in a subcutaneous xenograft model. Under DOX or DTX, tumors in the sh-ARTS group exhibited greater reductions in volume and weight than sh-Ctrl, whereas coknockdown (sh-ARTS + sh-Livin) blunted this therapeutic response (Fig. 5G and H and Fig. S10C and D). Importantly, ATG7 knockdown or CQ cotreatment diminished the growth advantage of the sh-ARTS + sh-Livin group during chemotherapy, indicating a requirement for intact autophagic flux.

Collectively, these findings indicate that ARTS promotes apoptosis-dependent autophagy and chemoresistance by suppressing Livin: Loss of Livin phenocopies ARTS activity by augmenting autophagy and undermining chemotherapy efficacy.

### Livin modulates autophagy and chemoresistance through the MDM2–p53 pathway in breast cancer cells

**Fig. 6.**
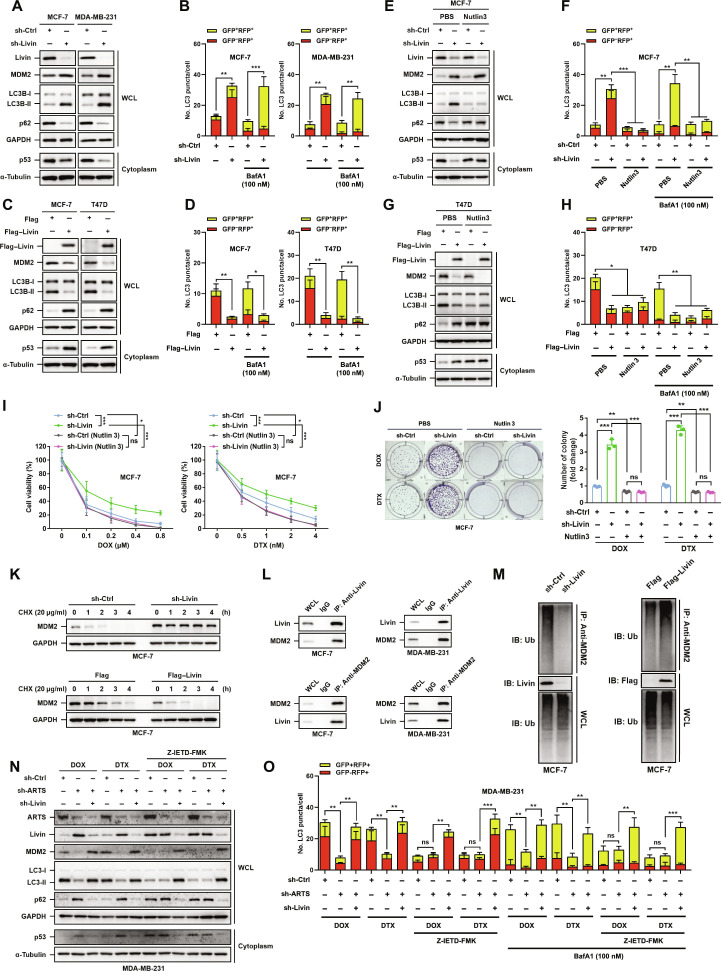
Livin regulates autophagy and chemoresistance through the MDM2–p53 pathway. (A and C) Immunoblot from MCF-7 and MDA-MB-231 with sh-Livin versus sh-Ctrl (A) and from MCF-7 and T47D with Flag–Livin versus Flag (C). WCLs and cytoplasmic fractions show reciprocal changes in MDM2, p53, LC3B, and p62. (B and D) mRFP–GFP–LC3 flux quantification. (B) sh-Ctrl versus sh-Livin in MCF-7 and MDA-MB-231; (D) Flag versus Flag–Livin in MCF-7 and T47D. BafA1 (100 nM, 3 h) where indicated. (E to H) Nutlin 3 (MDM2–p53 inhibitor) reverses Livin-dependent effects. (E and G) Immunoblot in MCF-7 (E) or T47D (G) with PBS versus Nutlin 3. (F and H) Corresponding LC3 reporter quantifications. (I) MTT curves of MCF-7 sh-Ctrl versus sh-Livin with or without Nutlin 3 under DOX (left) or DTX (right). (J) Colony formation of MCF-7 sh-Ctrl versus sh-Livin ± Nutlin 3 under DOX/DTX; quantification shown at right. (K) CHX chase in MCF-7 sh-Livin or Flag–Livin cells showing Livin-dependent MDM2 turnover. (L) Endogenous co-IP reveals a Livin–MDM2 complex in MCF-7 and MDA-MB-231. (M) Ubiquitination (Ub) assays: Livin depletion decreases, whereas Flag–Livin increases, MDM2 polyubiquitination. (N and O) Pathway integration. In MDA-MB-231, sh-ARTS (±sh-Livin or Z-IETD-FMK) alters Livin/MDM2/p53 and LC3/p62 as shown (N); LC3 reporter quantification for the same conditions (O). *P < 0.05; **P < 0.01; ***P < 0.001.

We first examined whether Livin connects to the MDM2–p53 module that controls autophagy. Livin depletion in MCF-7 and MDA-MB-231 cells increased MDM2 and reduced p53 and p62, accompanied by an LC3-I→LC3-II conversion; conversely, Flag–Livin overexpression lowered MDM2 and raised p53 and p62 while suppressing LC3 conversion (Fig. 6A and C). Tandem mRFP–GFP–LC3 reporters corroborated these biochemical readouts: Livin knockdown increased puncta, whereas Livin overexpression reduced puncta, and these changes persisted in the presence of BafA1 (Fig. 6B and D and Fig. S11A to D).

To test dependency on the MDM2–p53 interaction, we used Nutlin 3, which disrupts MDM2 binding to p53. Nutlin 3 restored p53 and p62 and reversed LC3 changes in Livin-depleted MCF-7 cells (Fig. 6E to H and Fig. S11E and F). Functionally, Nutlin 3 rescued the loss of DOX/DTX cytotoxicity caused by Livin knockdown (Fig. 6I and J and Fig. S11G and H). These data place Livin upstream of MDM2-dependent control of p53 and autophagy.

To understand how Livin regulates MDM2, we performed cycloheximide (CHX) chase assays in MCF-7 cells (Flag–Livin, sh-Livin, and respective controls). Livin expression facilitated MDM2 degradation when protein synthesis was blocked by CHX (Fig. 6K). In addition, co-IP of endogenous proteins showed a Livin–MDM2 interaction in MCF-7 and MDA-MB-231 cells (Fig. 6L). Ubiquitination of MDM2 was reduced by Livin knockdown and increased by Livin overexpression in MCF-7 cells (Fig. 6M). Together, these results indicate that Livin promotes MDM2 ubiquitination and turnover, thereby restraining MDM2 activity and sustaining p53.

We next integrated the ARTS pathway with chemotherapy-induced autophagy. In MDA-MB-231 cells, DOX or DTX increased LC3 puncta in sh-Ctrl cells, whereas ARTS knockdown markedly attenuated this autophagic response, with immunoblotting showing reduction in LC3-II levels, and this was accompanied by decreased MDM2 and increased Livin, p62, and cytosolic p53; cosilencing Livin largely blocked these changes, while the caspase-8 inhibitor Z-IETD-FMK suppressed LC3 puncta and abolished differences between sh-Ctrl and sh-ARTS cells (Fig. 6N and O and Fig. S12A). In MCF-7/DOX cells, ARTS depletion similarly reduced DOX-induced LC3 puncta and induced corresponding changes in Livin, MDM2, LC3-II, p62, and p53, which were recapitulated by Z-IETD-FMK (Fig. S12B and C). Together, these data indicate that ARTS promotes chemotherapy-induced, caspase-8-dependent autophagy in part by restraining Livin and maintaining MDM2, thereby limiting sustained p53 accumulation under DOX/DTX.

Chemotherapy exerted drug- and cell-line-specific effects on p53. DOX robustly increased cellular p53 protein, whereas DTX had little effect (Fig. S13A). Concordantly, ARTS and canonical p53 target transcripts (DNA damage regulated autophagy modulator 1 (*DRAM1*), sestrin 1 (*SESN1*), sestrin 2 (*SESN2*), and DNA damage inducible transcript 4 (*DDIT4*)) were induced only in MCF-7 upon DOX but remained largely unchanged under DTX or in MDA-MB-231 (Fig. S13B, C, and E to H). Chromatin IP (ChIP)-quantitative real-time polymerase chain reaction (qPCR) showed p53 enrichment at the ARTS promoter in MCF-7, with no enrichment in MDA-MB-231, consistent with differential p53 stabilization and the transcription-defective tumor protein p53 (*TP53*) status of MDA-MB-231 (Fig. S13D). Moreover, in HCC1937 cells lacking functional p53, ectopic expression of p53-WT or the transcription-inactive mutant p53-R175H followed by chemotherapy still altered autophagic flux (Fig. S13I and J). Together, these data indicate that DOX-stabilized p53 is transcriptionally competent to engage ARTS in MCF-7, whereas mutant p53 in MDA-MB-231 cannot activate the ARTS promoter. However, this does not alter the autophagy and drug-response programs described above: The pathway relies primarily on the cytosolic pool of p53 rather than on its nuclear, transcriptional activity.

Collectively, these findings suggest that ARTS induces apoptosis-dependent, autophagy-mediated chemoresistance in breast cancer cells by regulating the MDM2–p53 pathway through suppression of Livin.

### Correlation between Livin/MDM2 and ARTS in breast cancer tissues and its role in chemoresistance

**Fig. 7.**
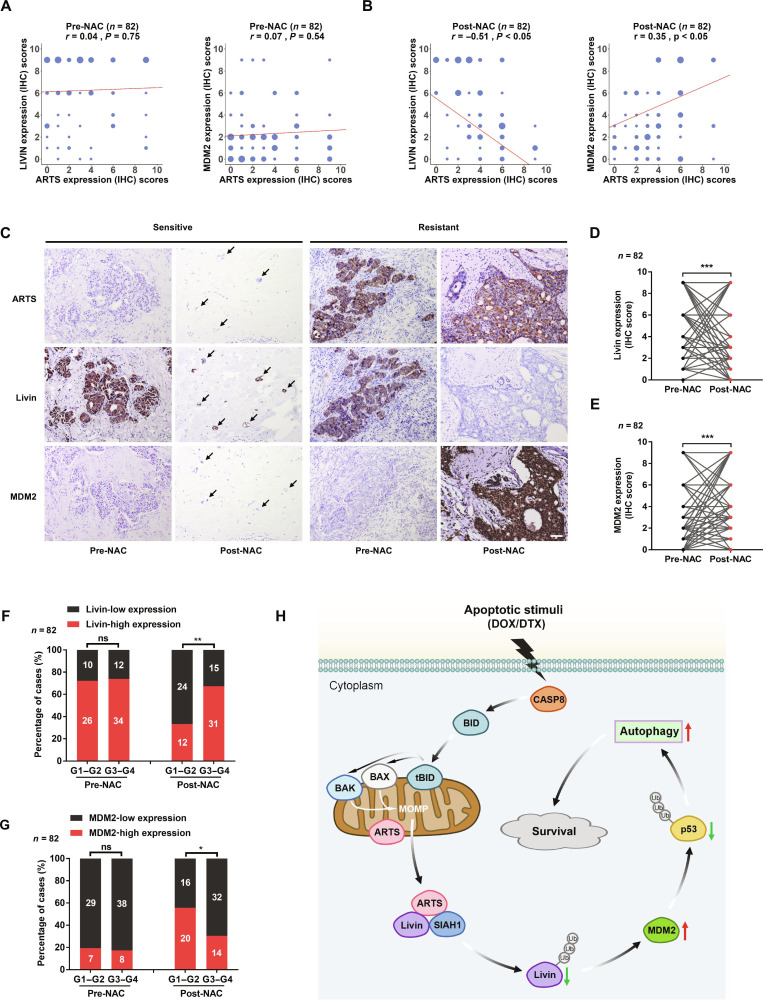
Correlation between ARTS/Livin/MDM2 expression and chemoresistance. (A and B) Spearman’s rank correlations between ARTS and Livin/MDM2 in 82 pre- and post-NAC breast cancer samples by IHC. (C) Representative IHC images of ARTS, Livin, and MDM2 in pre- and postchemotherapy samples of NAC-sensitive and NAC-resistant patients. The black arrow indicates a residual small cluster of tumor cells. Scale bar, 50 μm. (D and E) Changes in Livin and MDM2 protein levels in paired pre- versus post-NAC samples. (F and G) Associations between Livin/MDM2 protein and NAC response (MPG) in pre- and post-NAC samples. (H) Schematic model. Under apoptotic stimuli from DOX/DTX, caspase-8 (CASP-8) is activated to cleave BID to tBID, promoting MOMP and release of ARTS from mitochondria into the cytosol. Cytosolic ARTS recruits SIAH1 to ubiquitinate and degrade Livin, relieving its restraint on MDM2; enhanced MDM2 promotes ubiquitination and degradation of p53. Reduced p53 increases autophagy, supporting cell survival and chemoresistance. *P < 0.05; **P < 0.01; ***P < 0.001.

The expression of Livin and MDM2 in paired breast cancer tissues pre- and post-NAC was examined by IHC. Spearman’s rank correlation revealed a significant correlation between Livin/MDM2 and ARTS protein expression post-NAC, but not pre-NAC (Fig. 7A to C). Compared with pre-NAC cancers, post-NAC resistant tumors exhibited a significant decrease in Livin and a significant increase in MDM2 (Fig. 7D and E).

We next analyzed associations between Livin and MDM2 and clinicopathological features (MPG score, Ki-67, age, lymph node status, tumor–node–metastasis (TNM) stage, ER, PR, and HER2) in pre- and post-NAC samples. In post-NAC tumors, but not pre-NAC, lower Livin and higher MDM2 were associated with lower MPG scores (poorer pathological response) (Fig. 7F and G and Tables S3 and S4).

Together, these findings demonstrate a post-NAC correlation between Livin/MDM2 and ARTS and are consistent with an ARTS-mediated regulation of the Livin/MDM2 axis that contributes to chemoresistance in clinical specimens.

## Discussion

Classical proapoptotic molecules such as caspase-3 can, in specific contexts, exhibit prosurvival effects [10]. In the case of ARTS, it translocates into the cytosol during apoptosis, binds XIAP, and facilitates caspase activation [22,23]. Here, we show that, following exposure to chemotherapeutic agents, ARTS increases autophagy in an apoptosis-dependent manner, shifting cell fate toward survival. Although this may seem paradoxical at first glance [10], apoptosis and autophagy are intertwined programs that, together, determine cell fate [24–26]. For example, XIAP, a potent antiapoptotic inhibitor of apoptosis protein (IAP), restrains autophagy via the XIAP–MDM2–p53 pathway [27,28]. Beclin-1 regulates autophagy by engaging the antiapoptotic multidomain proteins BCL-2 and BCL-xL through its Bcl-2 homology 3 (BH3) domain [29]. Thus, the finely tuned interplay between apoptosis and autophagy is essential for maintaining the balance between survival and death in both physiological and pathological states.

NAC is administered before surgery to downsize or downstage locally advanced tumors, thereby improving the effectiveness of local therapies [4]. It is widely used in breast cancer, yet a subset of patients fails to respond to NAC because of drug resistance [30]. Comparative analyses of pre- versus post-NAC specimens can therefore yield clues to the mechanisms of NAC resistance. In parallel with advances in cancer biology, recent progress in machine learning has markedly improved biomedical image analysis and subject-specific modeling [31,32]. In our cohort, ARTS protein expression was significantly increased in post-NAC resistant tumors. ARTS is generally regarded as a key promoter of mitochondrial apoptosis [17]. Mechanistically, ARTS induces apoptosis by directly binding and inhibiting XIAP and BCL-2 and by facilitating p53-dependent, ubiquitin-mediated degradation of BCL-xL at mitochondria [18,33]. Consequently, ARTS has long been implicated in tumor initiation and progression [22]. However, our current data show that higher ARTS expression correlates negatively with patient survival in breast cancer, and, functionally, ARTS promotes chemoresistance in breast cancer cells.

Autophagy in cancer plays a dual, context-, and stage-dependent role, acting as either a suppressor or a promoter of tumor progression [34,35]. Notably, autophagy is strongly implicated in therapeutic resistance. In breast cancer, multiple regulatory axes have been reported, including the T-box transcription factor 15 (TBX15)–microRNA-152 (miR-152)–kinesin family member 2C (KIF2C)–pyruvate kinase M2 (PKM2) pathway contributing to DOX resistance [36], the long noncoding RNA OTUD6B antisense RNA 1 (OTUD6B-AS1)–miR-26a-5p–metadherin (MTDH) pathway conferring DTX resistance [37], and the hsa_circ_0092276–miR-348–ATG7 pathway mediating DOX resistance [38]. Here, we show that DOX/DTX induces MOMP and drives ARTS relocalization from mitochondria into the cytosol, thereby increasing autophagy in breast cancer cells. Consistent with prior reports that both extrinsic (death receptor) and intrinsic (mitochondrial) pathways contribute to drug-induced apoptosis, DOX can up-regulate tumor-necrosis-factor-related apoptosis-inducing ligand (TRAIL) and Fas ligand to activate caspase-8 [39]; in our system, chemotherapy likewise activated caspase-8. Pharmacologic inhibition of caspases or autophagy attenuated ARTS-mediated chemoresistance. Thus, our work identifies a previously unrecognized role for ARTS in promoting autophagy in an apoptosis-dependent manner to drive chemoresistance in breast cancer.

As previously reported, Livin has been linked to autophagy and reduced gemcitabine chemosensitivity in bladder cancer [40]. In colon cancer cells, Livin promotes autophagy and decreases sensitivity to 5-fluorouracil through an apoptosis/autophagy-dependent mechanism [41]. In renal carcinoma cells, Livin silencing increases cisplatin chemosensitivity [42]. These indicate that Livin plays a context- and tissue-dependent role in autophagy, apoptosis, and drug response, a notion further supported by evidence for a dual role of Livin in tumorigenicity under different conditions [43]. In our study, upon exposure to DOX or DTX, ARTS directly interacted with Livin in the cytosol in an apoptosis-dependent manner, mediated by binding of the C-terminal 68 amino acids of ARTS to the BIR domain of Livin. This interaction promoted SIAH1-dependent ubiquitination and proteasomal degradation of Livin, analogous to the previously described ARTS–SIAH1–XIAP pathway [15]. Livin is regulated at multiple layers (transcription factors, stress signaling, microRNAs, translation, and proteostasis), and ARTS represents a branch that directly controls Livin protein stability. Functionally, Livin constrained autophagy and reduced chemoresistance in our breast cancer models; accordingly, ARTS promoted apoptosis-dependent autophagy and chemoresistance at least in part via suppression of Livin.

We previously reported that XIAP regulates autophagy via the MDM2–p53 axis [27]. In the current study, we show that Livin negatively regulates MDM2 and positively regulates p53. The MDM2–p53 pathway is critically involved in cancer initiation and progression, and inhibiting the MDM2–p53 interaction is an effective therapeutic strategy [44,45]. Nutlin 3, a potent MDM2 antagonist, stabilizes p53 by disrupting its interaction with MDM2 and has shown promise in preclinical cancer models [27]. Our findings suggest that targeting this axis may help restore apoptotic sensitivity in chemoresistant tumors. Because the MDM2–p53 pathway regulates both apoptosis and autophagy, these data provide a rationale for combining chemotherapy with Nutlin 3. Notably, the effects of the ARTS–Livin–MDM2–p53 pathway on autophagy and chemoresistance were attenuated when apoptosis was inhibited with Z-IETD-FMK. Thus, DOX and DTX trigger apoptosis and promote ARTS relocalization from mitochondria to the cytosol in breast cancer cells, where ARTS regulates autophagy through a Livin/MDM2/p53-dependent mechanism, ultimately driving chemoresistance. This ARTS/Livin/MDM2/p53 axis appears to be primarily involved in acquired chemoresistance of breast cancer cells. Consistent with a context-dependent model, the pathway is expected to operate in tumors that retain functional p53, exhibit MDM2 activity, and express Livin; its impact diminishes when p53 is defective or Livin is scarce.

In summary, chemotherapeutic agents such as DOX and DTX activate caspase-8, cleaving BID to tBID and engaging Bax/Bak-dependent MOMP. This apoptotic priming drives relocalization of ARTS from mitochondria into the cytosol (Fig. 7H). In the cytosol, ARTS binds Livin and recruits the E3 ligase SIAH1, promoting ubiquitin-mediated proteasomal degradation of Livin. Loss of Livin releases its restraint on MDM2, thereby enhancing MDM2-mediated ubiquitination and degradation of p53. The resulting reduction in p53 promotes autophagy, enabling tumor cell survival and contributing to chemoresistance. Collectively, these findings delineate an apoptosis-dependent pathway whereby chemotherapeutic stress initiates mitochondrial apoptosis, activates the ARTS–Livin–MDM2–p53 axis, and drives prosurvival autophagy, ultimately fostering breast cancer chemoresistance.

## Materials and Methods

### Patients and tissue samples

Human breast cancer tissue samples were obtained from the First Affiliated Hospital of Anhui Medical University (Hefei, Anhui, China). Clinicopathological information was retrieved from the Department of Pathology. Between 2014 and 2019, 92 paired specimens (pre- and post-NAC) were collected from patients receiving DOX- or DTX-based NAC: pre-NAC core needle biopsies and post-NAC surgical resections, respectively, with postoperative histologic response evaluated [30]. All cases were assessed independently by 2 pathologists blinded to the study, according to the MPG system and standard criteria [46]. G1 and G2 were considered resistant, whereas G3 to G5 were considered sensitive [47]. Because G5 denotes complete absence of malignant cells in tumor site sections, the 10 G5 cases were excluded from downstream analyses, leaving 82 of 92 cases for IHC assessment. Written informed consent was obtained from all participants. The study was approved by the Institutional Review Board (approval no. 83230247) prior to initiation.

### Bioinformatic analyses

RNA-seq datasets generated in this study have been deposited in the GEO under accession GSE288073 and are publicly accessible. Apoptosis-related and mitochondria-related gene sets were obtained from UniProt (https://www.uniprot.org). The association between ARTS mRNA expression and survival in breast cancer was analyzed using Kaplan–Meier plotter (https://kmplot.com). Alteration frequencies of ARTS and IAP genes were assessed via cBioPortal for Cancer Genomics (https://www.cbioportal.org).

### Immunohistochemistry

IHC was performed as previously described [48]. Primary antibodies are listed in Table S5. For each specimen, 5 random high-power fields were examined. Staining intensity was scored 0 to 3 (0, none; 1, weak; 2, moderate; 3, strong). The percentage of positive tumor cells was scored 0 to 3 (0, none; 1, <25%; 2, 25% to 50%; 3, >50%). The final IHC score was calculated as intensity × percentage [49]. Scores 0 to 3 were classified as low expression, and scores >3 were classified as high expression.

### Cell culture and treatment

Human breast cancer cell lines (MCF-7, BT549, SUM149, HCC1937, SUM159, SKBR3, MDA-MB-468, T47D, BT474, and MDA-MB-231) and HEK293 cells were obtained from American Type Culture Collection and cultured under supplier-recommended conditions. DOX-resistant MCF-7 cells were generated in-house by stepwise selection with increasing concentrations of DOX [50]. Reagents are listed in Table S6. Cell lines were authenticated by short tandem repeat profiling; routine testing confirmed absence of mycoplasma contamination. No cell line was passaged more than 30 times.

### Cell viability assay

Cell viability was measured as previously described [30]. Briefly, cells were seeded into 96-well plates (Corning, 3799) and allowed to adhere. After the indicated treatments, 100 μl of 3-(4,5-dimethylthiazol-2-yl)-2,5-diphenyltetrazolium bromide (MTT) reagent (Sangon Biotech, A600799) was added to each well, and cells were incubated for 2 h. The supernatant was then removed, 100 μl of dimethyl sulfoxide (DMSO; Sangon Biotech, A610163) was added to dissolve the formazan, and absorbance was measured at 570 nm.

### Colony formation assay

Colony formation assay was carried out as previously described [30]. Briefly, cells were seeded into 6-well plates (Corning, 3516) and subjected to the indicated treatments. After 2 weeks of culture, colonies were washed twice with PBS, fixed in methanol, and stained with 0.1% crystal violet (Sangon Biotech, A600331).

### Cells transfection and lentivirus generation

Cell transfection and lentiviral production were performed following protocols described previously [30]. The mammalian expression vector pSin was used to construct ARTS, Livin, and SIAH1 expression plasmids. For RNA interference, pLKO.1 was used to clone shRNA constructs. shRNAs were obtained from The RNAi Consortium (MISSION TRC shRNA library, Sigma-Aldrich); oligonucleotide sequences are listed in Table S7. Stable cell lines were generated by lentiviral transduction, followed by puromycin selection (Sangon Biotech, A606719).

### Tumor xenograft mouse models

Four-week-old female BALB/c nude mice were obtained from Beijing Vital River Laboratory Animal Technology and housed in accordance with protocols approved by the Institutional Animal Care and Use Committee. This study was approved by the Institutional Animal Care and Ethics Committee of Anhui Medical University (approval no. 20230660).

For tumor implantation, a cell suspension was orthotopically injected into the mammary fat pad (3 × 10^6^ tumor cells per site) in an equal volume of Matrigel (BD Biosciences, 356234; 8 to 10 mice per group were used, whereas supplementary experiments included 5 to 6 mice per group). Tumor growth was monitored once weekly for up to 7 weeks. Beginning in week 2, when mean tumor volume reached ~50 mm^3^, mice were randomized to receive intraperitoneal injections of PBS (vehicle), DOX (2.5 mg/kg), DTX (5 mg/kg), CQ (40 mg/kg; autophagy inhibitor), or Z-IETD-FMK (10 mg/kg), twice per week, as indicated (reagents listed in Table S6).

Tumor dimensions were measured with calipers, and volumes were calculated as follows: volume (mm^3^) = (width^2^ × length) / 2. Mice were euthanized 7 weeks postimplantation; primary tumors were collected and fixed in 10% neutral-buffered formalin for subsequent IHC.

### Immunofluorescence and colocalization analysis

Cells were plated on glass coverslips (Biosharp, BS-14-RC) and treated as indicated. Samples were fixed in 4% paraformaldehyde for 15 min at room temperature, washed in PBS, permeabilized with 0.1% Triton X-100 (Sangon Biotech, A600198) for 10 min, and blocked in 2% bovine serum albumin (Sangon Biotech, A602440) for 1 h. Primary antibodies (listed in Table S5) were incubated overnight at 4 °C. After PBS washes, Alexa-Fluor-conjugated secondary antibodies (Thermo Fisher Scientific, A-11008/A-11005) were applied for 1 h at room temperature in the dark; nuclei were counterstained with 4′,6-diamidino-2-phenylindole (DAPI; Sangon Biotech, E607303).

Images were acquired on a Zeiss LSM 880 confocal microscope using identical laser power, detector gain, and pinhole settings across conditions. Colocalization was quantified with the Zeiss ZEN built-in colocalization module, and the PCC was computed on a per-cell basis. For each condition, ≥30 cells were analyzed, and results are reported as means ± SEM of per-cell PCC values.

### JC-1 assay by flow cytometry

ΔΨm was assessed by JC-1 staining, followed by flow cytometry: Cells were incubated with JC-1 (2 μM, 20 to 30 min, 37 °C, dark), washed, harvested, and acquired on a 488-nm cytometer (green/fluorescein isothiocyanate [FITC], ~530/30; red/phycoerythrin [PE], ~585/42). Debris and doublets were excluded; ≥10,000 live singlets were recorded per sample. Red/green mean fluorescence intensity ratios (PE/FITC) and % low-ΔΨm (gate defined by carbonyl cyanide *m*-chlorophenyl hydrazone (CCCP)-treated controls) were quantified in FlowJo using identical photomultiplier tube/compensation across conditions.

### Tandem mRFP–GFP–LC3 autophagic flux assay

We generated stable cell lines expressing tandem mRFP–GFP–LC3. Where indicated, BafA1 (100 nM, 3 h) was added before imaging. Confocal images were acquired under identical acquisition parameters. For flux quantification, GFP^+^RFP^+^ (yellow, autophagosomes) and RFP^+^ only (red, autolysosomes) puncta were counted per cell. For each condition, 30 randomly selected cells were analyzed per experiment, and data are reported as puncta per cell (means ± SEM).

### Immunoblot analysis

Immunoblot analysis was performed as previously described [30]. The primary antibodies are listed in Table S5.

### Coimmunoprecipitation

Co-IP was carried out as previously described [51]. Briefly, cells were lysed in radioimmunoprecipitation assay (RIPA) buffer (Beyotime, P0013C) supplemented with complete protease inhibitor cocktail (Sigma-Aldrich, 11836170001). After preclearing with Protein A/G PLUS-Agarose beads (Santa Cruz Biotechnology, sc-2003) for 1 h at 4 °C, primary antibodies (Table S5) were added to the whole-cell lysates (WCLs) and incubated overnight at 4 °C with rotation. Beads were then added and incubated for 1 h. Immune complexes were washed 5× with RIPA buffer and analyzed by immunoblotting.

### Mass spectrometry

Proteins were reduced with dithiothreitol (Sangon Biotech, A620058) at 37 °C for 2 h and then alkylated with iodoacetamide (Sangon Biotech, A600539) for 30 min at room temperature in the dark. Samples were digested with trypsin (Sangon Biotech, A003702) at a 1:50 (w/w) enzyme-to-substrate ratio overnight at 37 °C, followed by a second trypsin addition at the same ratio for 4 h. Peptides were desalted using a solid-phase extraction column, dried, and subjected to LC-MS/MS on a Q Exactive HF mass spectrometer (Thermo Fisher Scientific) coupled to an UltiMate 3000 UHPLC system for peptide separation.

### Proximity ligation assay

Cells were seeded at low density onto round glass coverslips and fixed with 4% paraformaldehyde (Sangon Biotech, E672002) for 20 min at room temperature. Duolink In Situ PLA was performed according to the manufacturer’s instructions (Merck/Sigma-Aldrich, DUO92101) using the primary antibodies listed in Table S5. Images were acquired on a Zeiss LSM 880 confocal laser scanning microscope.

### Nuclear–cytoplasmic–mitochondrial protein separation assay

Nuclear, cytoplasmic, and mitochondrial fractions were prepared using the Nuclear and Cytoplasmic Protein Extraction Kit (Beyotime, P0027) and the Cell Mitochondria Isolation Kit (Beyotime, C3601) according to the manufacturers’ instructions.

### Ubiquitination assay

Cells were pretreated with MG132 (5 μM) for 12 h and then lysed in RIPA buffer (Beyotime, P0013C) supplemented with complete protease inhibitor cocktail (Sigma-Aldrich, 11836170001). Lysates were precleared with Protein A/G PLUS-Agarose beads (Santa Cruz Biotechnology, sc-2003) for 1 h at 4 °C, after which primary antibodies (Table S5) were added to the WCLs and incubated overnight at 4 °C with rotation. Beads were then added and incubated for 1 h. Immune complexes were washed 5× with RIPA buffer and analyzed by immunoblotting. Additional reagents are listed in Table S6.

### Protein stability assay

Cells were plated and grown to the desired confluency and then treated with CHX (20 μg/ml). Cells were harvested at the indicated time points (0, 1, 2, 3, and 4 h) and analyzed by immunoblotting. Reagents used are listed in Table S6.

### Quantitative real-time polymerase chain reaction

qPCR was performed as previously described [30]. Primer sequences are listed in Table S7.

### ChIP and ChIP-qPCR

Cells were cross-linked with 1% formaldehyde for 10 min at room temperature and quenched with 125 mM glycine for 5 min. After lysis, chromatin was sonicated on ice to ~200- to 500-bp fragments (30-s on/30-s off, 10 to 15 min; verified on agarose gel). Equal chromatin amounts were incubated overnight at 4 °C with anti-p53 antibody or species-matched immunoglobulin G (IgG) control, followed by capture with Protein A/G magnetic beads for 2 to 4 h. Beads were washed sequentially with low-salt, high-salt, LiCl, and tris–EDTA buffers. DNA–protein complexes were eluted and reverse-cross-linked at 65 °C overnight and then treated with ribonuclease A (37 °C, 30 min) and Proteinase K (55 °C, 1 h), and DNA was purified by spin columns.

ChIP-qPCR was performed using the same SYBR system as above with primers targeting the ARTS promoter (Table S7). Enrichment was calculated as %Input (100 × 2^Ct_input-Ct_IP^) and normalized to IgG and/or the negative region.

### Statistical analysis

Statistical analyses were performed using GraphPad Prism 7. Data are presented as means ± SD or means ± SEM as indicated. Depending on the experiment, Student’s *t* test, one-way analysis of variance (ANOVA), χ^2^ test, or log-rank (Mantel-Cox) test was used, as specified in the figure legends. *P* < 0.05 was considered statistically significant.

## Data Availability

Source data supporting the findings of this study are available from the corresponding author upon reasonable request.
