# Supplementary material for: ARTS Confers Chemoresistance of Breast Cancer by Inducing Apoptosis-Dependent Autophagy via Livin–MDM2–p53 Pathway
Source: Research (Wash D C). 2026 Jan 15;9:1086. doi: 10.34133/research.1086 (PMC12805590; doi:10.34133/research.1086)
Supplement: Supplementary 1 — Figs. S1 to S13 Tables S1 to S7 [file research.1086.f1.docx]

**ARTS confers chemoresistance of breast cancer by inducing apoptosis-dependent autophagy via Livin-MDM2-p53 pathway**

Hao Wang, Qianying Guo, Yuting Shen, Keshuo Ding, Yinfeng Chen, Xiaonan Wang, Xing Huang and Zhengsheng Wu

**Supplementary data**

These supplementary data contain 13 figures and 7 tables.

**
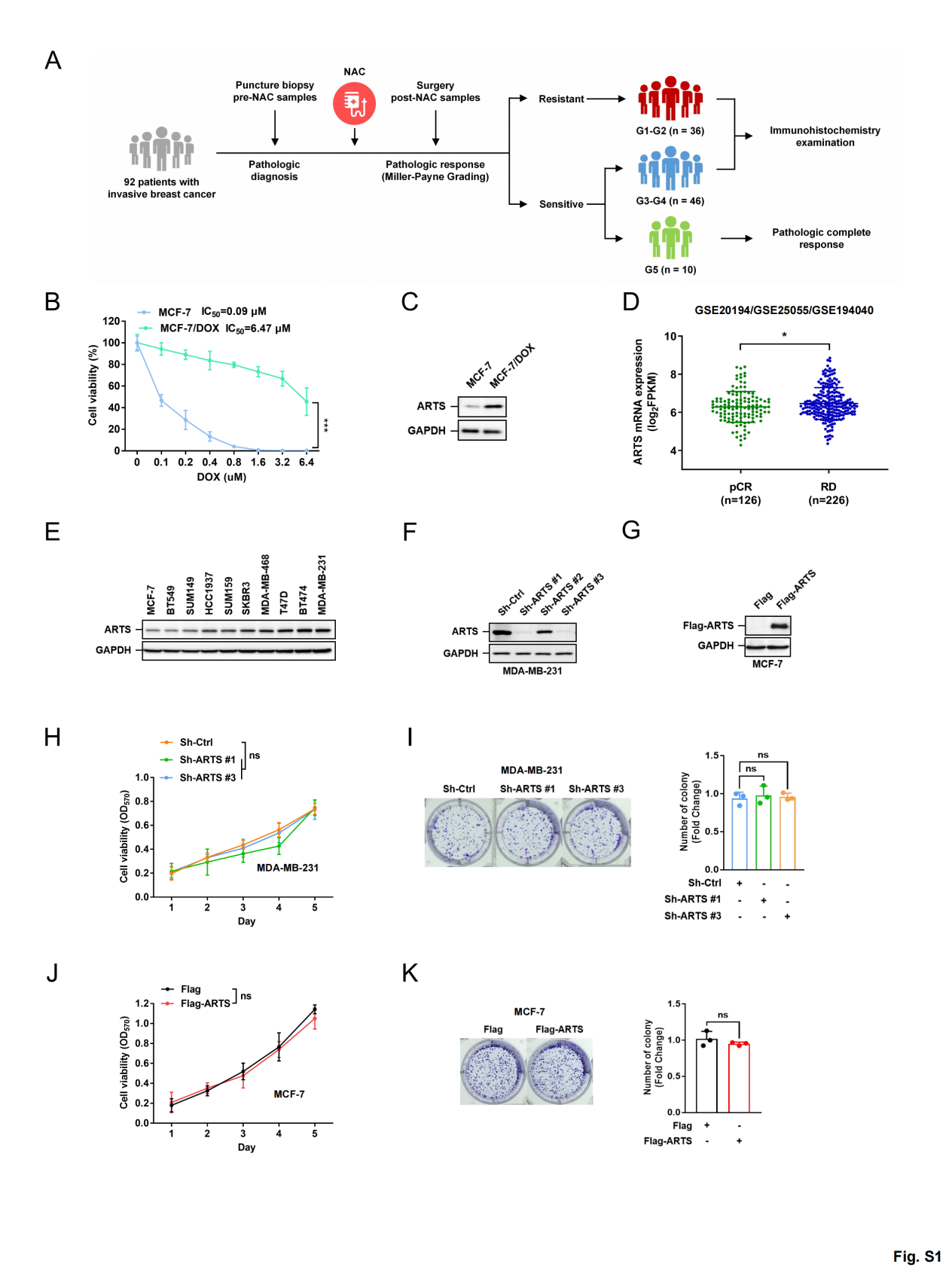
Supplementary Figure 1. ARTS indicates a worse prognosis and is upregulated in chemoresistant breast cancer cells. (A)** Sample collection: the study included 92 patients with breast cancer who received NAC before surgery. Both puncture biopsy samples and surgically resected tumor samples from the same patient were collected in pairs. Ten cases with G5 were excluded based on rigorous pathological evaluation. Patients were then classified into a resistant group (n = 36) and a sensitive group (n = 46). Pre- and post-NAC sample pairs were analyzed using immunohistochemistry (IHC). **(B)** MCF-7 and MCF-7/DOX cells were treated as indicated and MTT was performed. **(C)** ARTS protein expression in MCF-7 and MCF-7/DOX cells detected by immunoblot analysis. **(D)** Combined analysis of three neoadjuvant cohorts (GSE20194/25055/194040). **(E)** ARTS protein expression in breast cancer cell lines detected by immunoblot. **(F-K)** MTT and colony formation assays were performed on sh-Ctrl and sh-ARTS MDA-MB-231 cells, as well as Flag and Flag-ARTS MCF-7 cells.


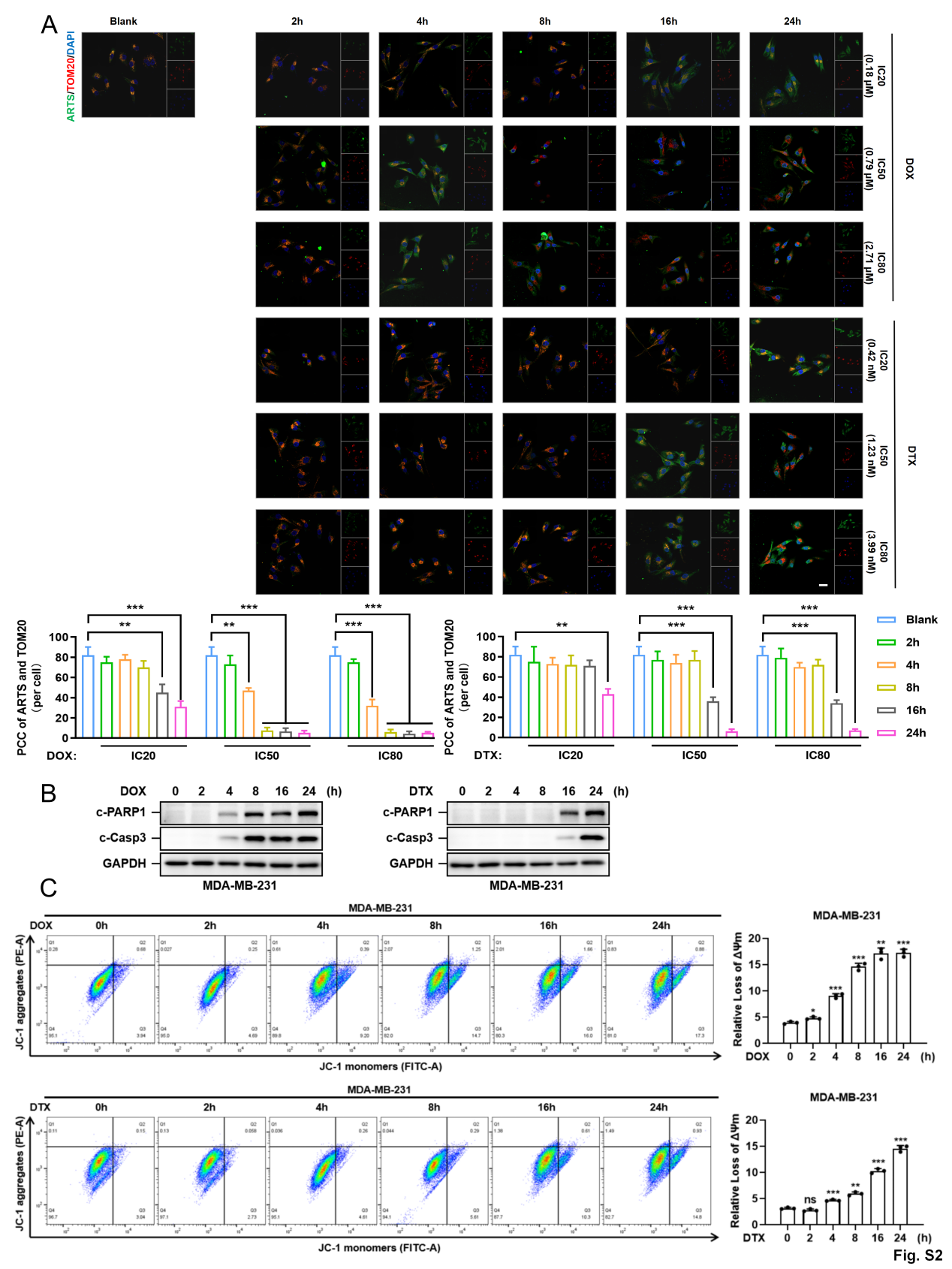
**Supplementary Figure 2. Time-course imaging, apoptosis markers, and ΔΨm measurements accompanying ARTS localization assays.** **(A)** Confocal immunofluorescence (IF) co-staining for ARTS and TOM20 in MDA-MB-231 cells was performed after treatment with chemotherapeutic agents across a range of doses (IC20-80) and time points (2, 4, 8, 16, 24h). Per-cell colocalization was quantified as Pearson’s correlation coefficient (PCC). Scale bar, 20 μm. **(B)** Immunoblot for apoptosis markers (e.g., cleaved PARP, cleaved caspase-3) were collected at the same time points. **(C)** JC-1 based flow cytometry quantified mitochondrial membrane potential (ΔΨm).


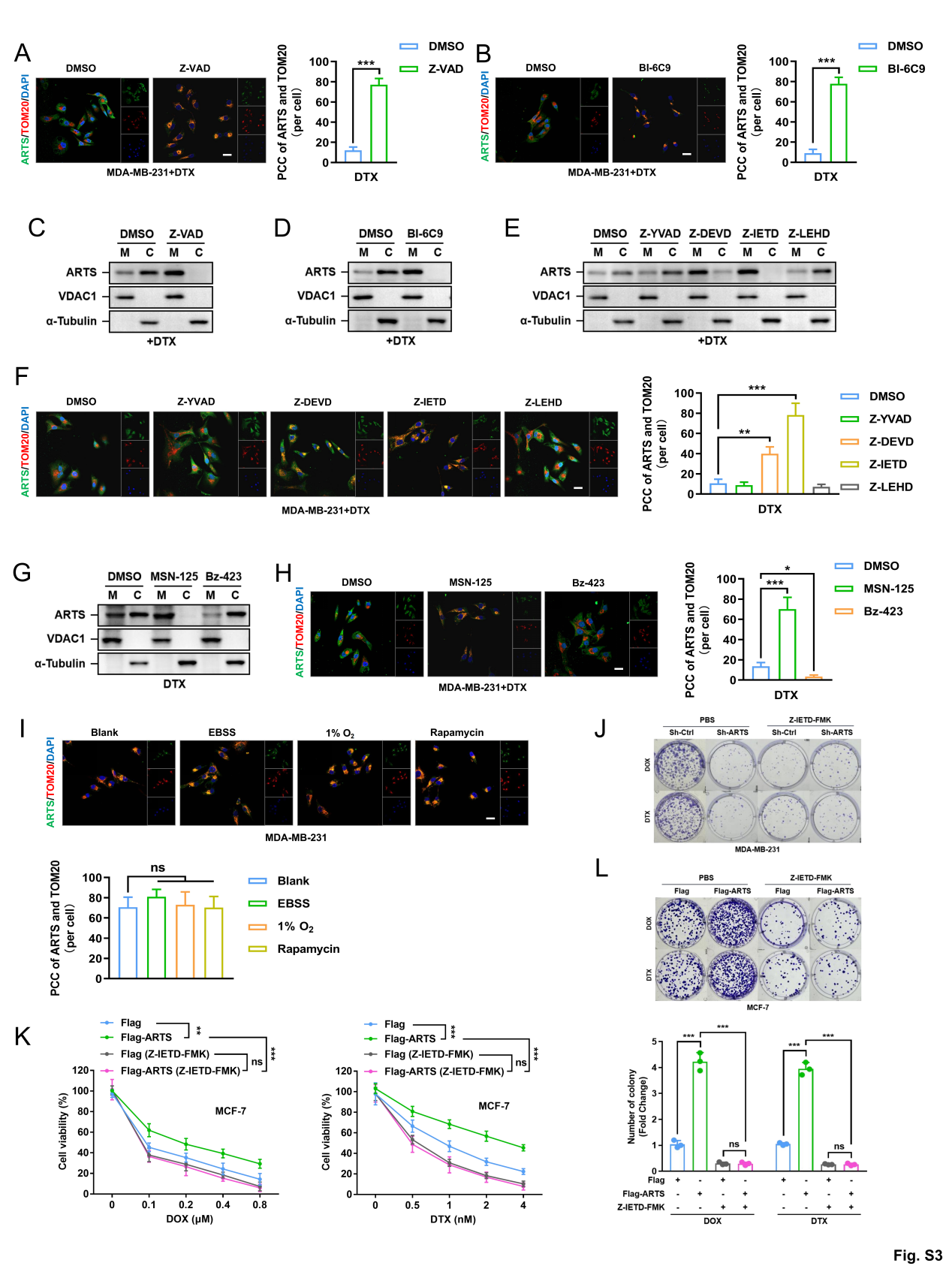
**Supplementary Figure 3****. DTX-focused replication and fractionation-based ARTS localization with pharmacologic dissection. (A-B)** IF of ARTS/TOM20 following DTX exposure was performed as in S2, with PCC analysis in ≥30 cells per condition. Scale bar, 20 μm. **(C-E)** Subcellular fractionation separated cytosolic and mitochondrial compartments in MDA-MB-231 cells. Cells were treated as indicated. **(F)** IF under selective caspase inhibitors (Z-YVAD, Z-DEVD, Z-IETD, Z-LEHD) with PCC analysis as above. Scale bar, 20 μm. **(G-H)** Fractionation or IF following DTX with MSN-125 (Bax/Bak inhibitor) or Bz-423 (Bax/Bak activator) in MDA-MB-231 cells. Scale bar, 20 μm. **(I)** Non-apoptotic stress controls included EBSS starvation, hypoxia (1% O_2_), and mTOR inhibition (Rapamycin). Scale bar, 20 μm. **(J)** Colony assays of sh-Ctrl and sh-ARTS MDA-MB-231 cells with DOX/DTX. **(K)** MTT assays in MCF-7 Flag/Flag-ARTS cells ± Z-IETD-FMK across DOX/DTX dose ranges. **(L)** Colony formation in MCF-7 cells treated as indicated.


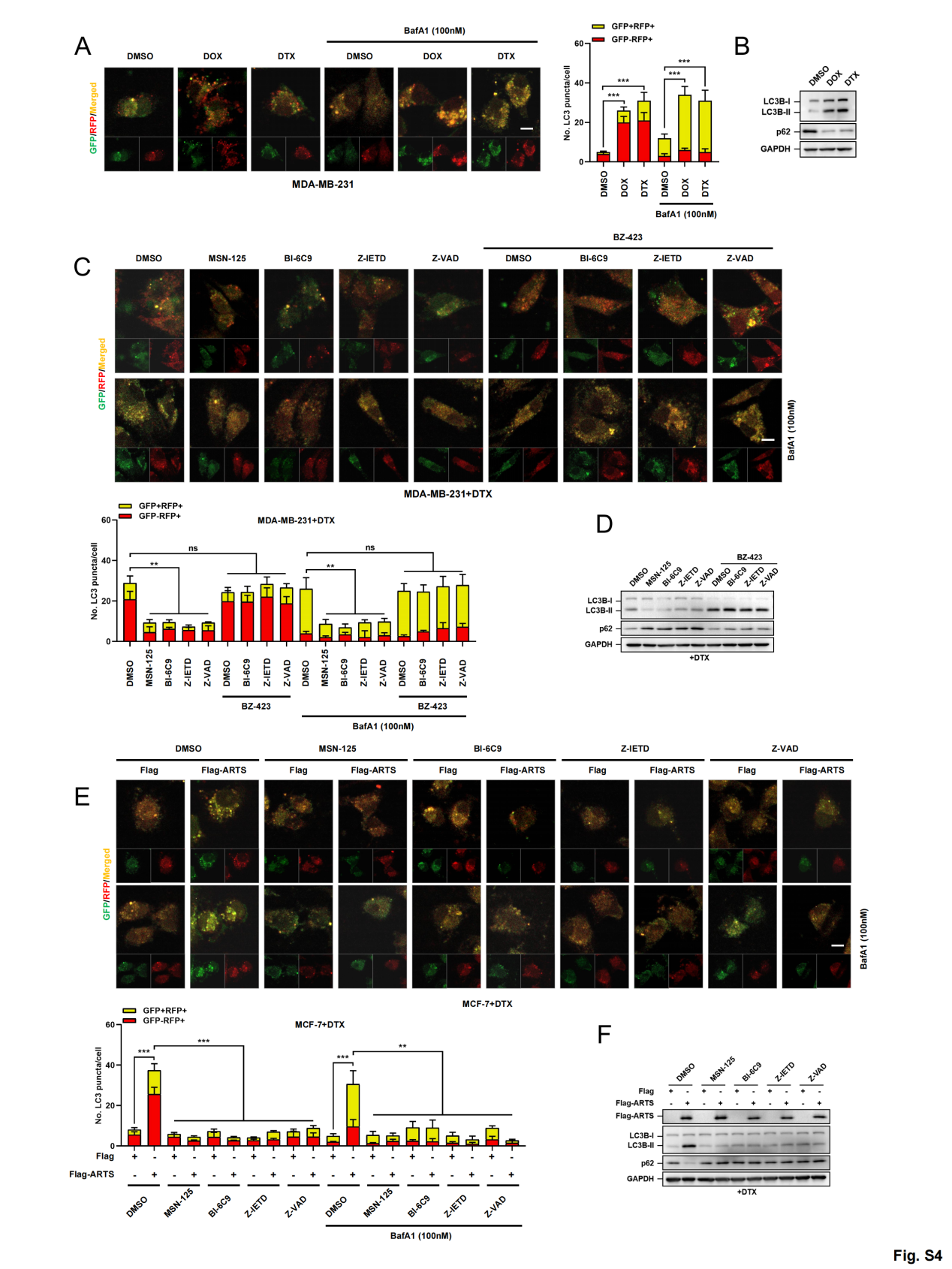


**Supplementary Figure 4. Autophagy-flux imaging and immunoblot workflows under chemotherapy, MOMP/caspase perturbation, and ARTS overexpression.**

**(A)** Tandem mRFP-GFP-LC3 reporter in MDA-MB-231 cells treated with vehicle, DOX or DTX with or without Bafilomycin A1 (BafA1, 100 nM, 3 h). Confocal acquisition used identical settings; GFP+RFP+ (yellow, autophagosomes) and RFP+ only (red, autolysosomes) puncta were counted per cell (≥30 cells/condition/experiment). Scale bar, 10 μm. **(B)** Whole-cell lysates from MDA-MB-231 cells collected after the same treatments were immunoblotted for LC3B-I/II and p62, with GAPDH as a loading control. **(C)** Reporter imaging in MDA-MB-231 cells exposed to Bz-423 or DTX together with the indicated pathway modulators (MSN-125, BI-6C9, Z-IETD-FMK, Z-VAD-FMK); where shown, BafA1 was added before imaging; puncta were quantified as in (A). Scale bar, 10 μm. **(D)** Parallel immunoblotting in MDA-MB-231 cells after DTX treatment in the presence of Bz-423 and the same modulators was performed for LC3B-I/II and p62, with GAPDH as a loading control. **(E)** Reporter imaging in MCF-7 cells expressing Flag or Flag-ARTS under DTX with the indicated inhibitors. Scale bar, 10 μm. **(F)** Corresponding immunoblots in MCF-7 cells for LC3B-I/II and p62 after DTX with Flag/Flag-ARTS and the same inhibitors.


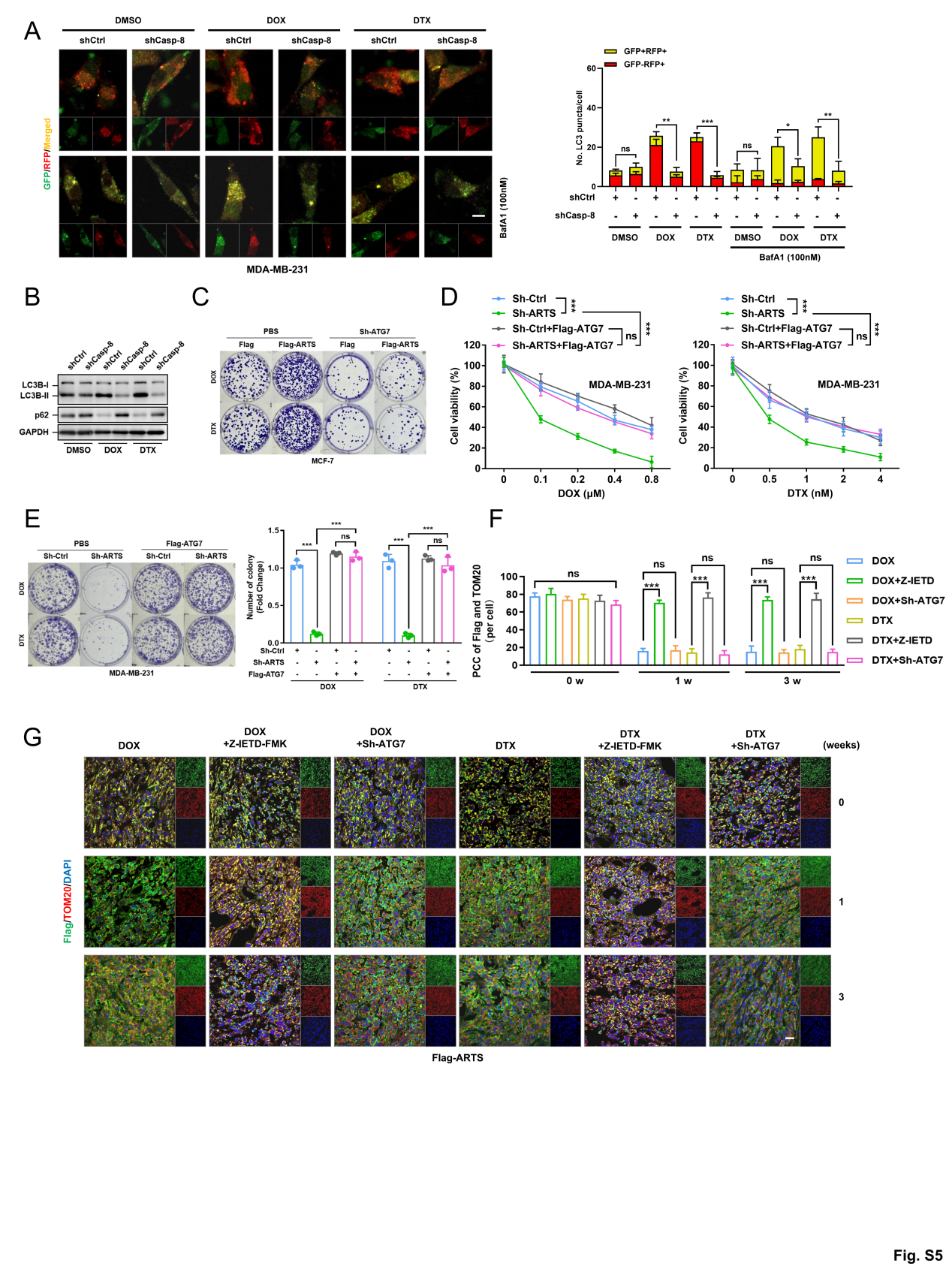


**Supplementary Figure 5. Caspase-8, ATG7, and in-vivo staining workflows linked to chemotherapy.**

**(A)** Tandem mRFP-GFP-LC3 imaging in MDA-MB-231 cells expressing shCtrl or shCasp-8 after DMSO/DOX/DTX treatment. Scale bar, 10 μm. **(B)** Whole-cell lysates from MDA-MB-231 cells subjected to the same treatments were immunoblotted for LC3B-I/II and p62, with GAPDH as a loading control. **(C)** Colony formation in MCF-7 cells (Flag or Flag-ARTS) with or without shATG7 under the indicated treatments. **(D)** MTT viability curves for MDA-MB-231 (shCtrl, shARTS, and combinations with Flag-ATG7) exposed to graded DOX/DTX. **(E)** Colony assays in MDA-MB-231 using the same genetic combinations and drug regimens. **(F-G)** *In vivo* tumor staining: orthotopic xenografts derived from MCF-7 Flag-ARTS were treated with DOX/DTX alone or combined with Z-IETD-FMK or shATG7 and harvested at 0, 1, and 3 weeks. Tumor sections were co-stained for Flag (green), TOM20 (red), and DAPI; images were acquired with identical parameters, and per-cell PCC of Flag/TOM20 was quantified from multiple fields per tumor (≥30 cells/field). Scale bar, 20 μm.


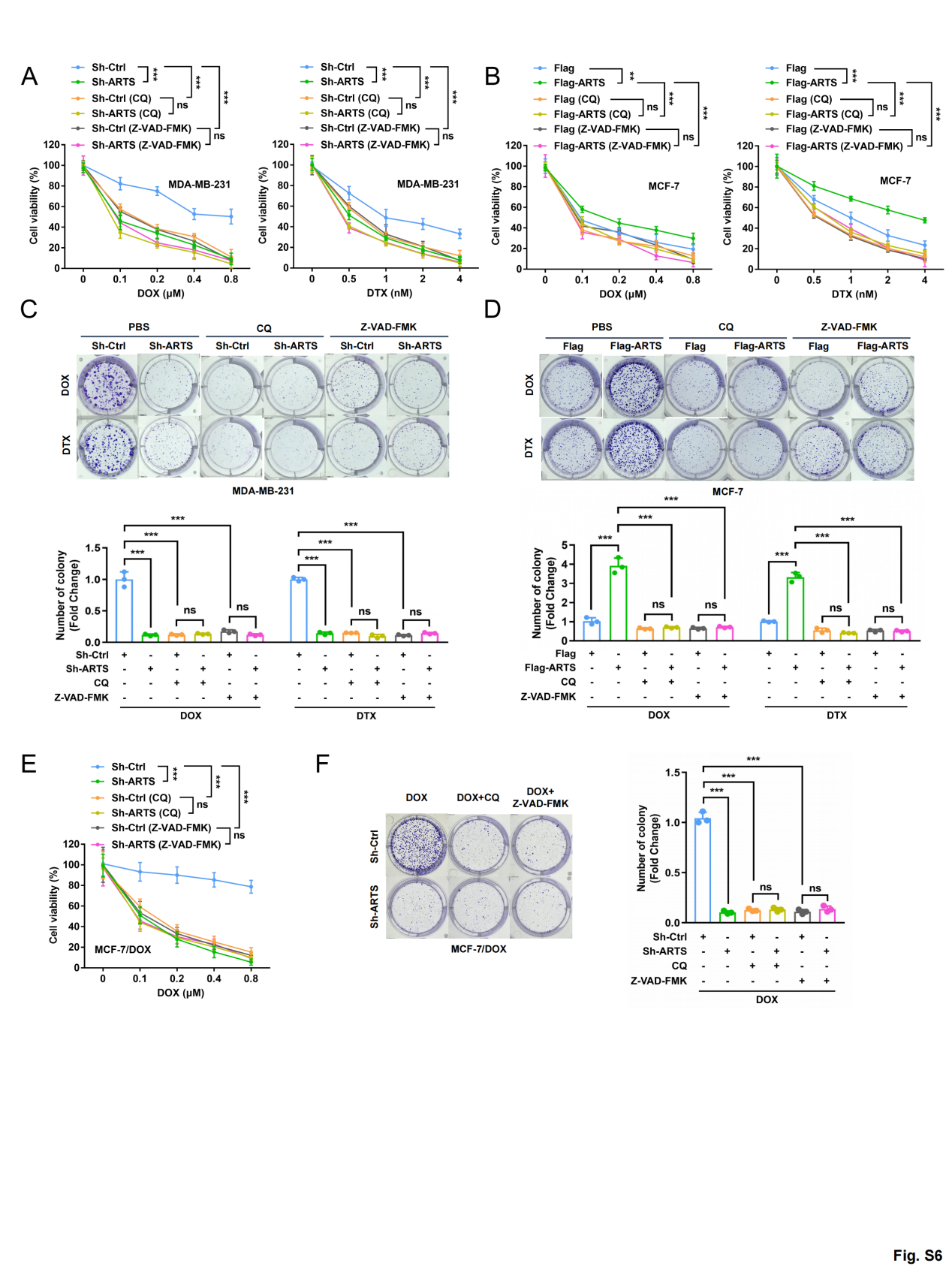


**Supplementary Figure 6. Inhibition of autophagy or apoptosis blocks ARTS-mediated chemoresistance in breast cancer cells. (A-B)** MTT and **(C-D)** colony formation assays were performed with sh-Ctrl and sh-ARTS MDA-MB-231 cells, as well as Flag and Flag-ARTS MCF-7 cells, then treated as indicated. **(E-F)** sh-Ctrl and sh-ARTS MCF-7/DOX cells were treated as indicated and MTT and colony formation assays were performed.

**
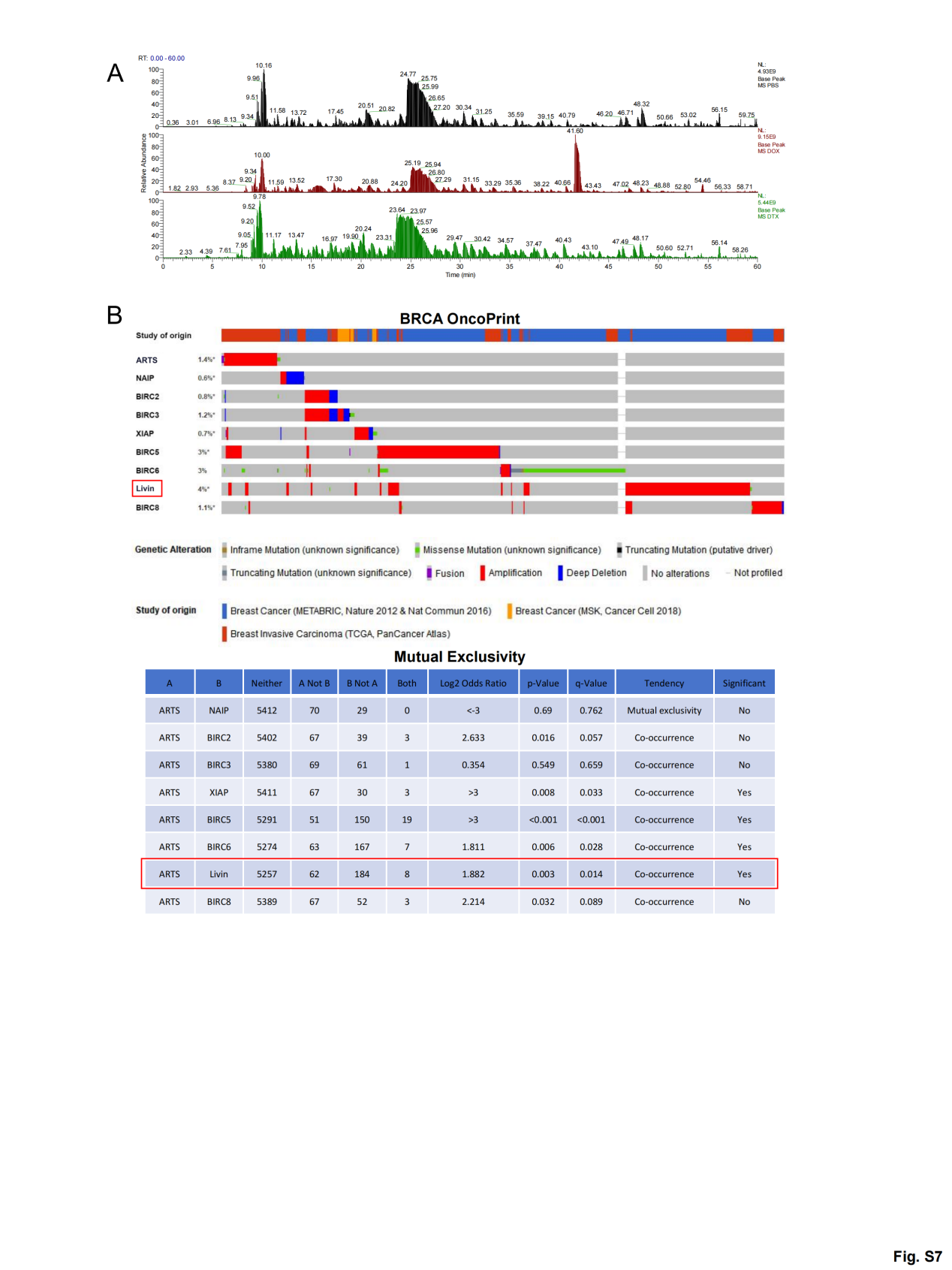
**

**Supplementary Figure 7. Screening of the downstream proteins of ARTS. (A)** Base peak chromatogram of mass spectrometry. **(B)** Analysis of ARTS and IAPs with the percentage of gene alterations in breast cancer performed using data from cBioPortal for Cancer Genomics.


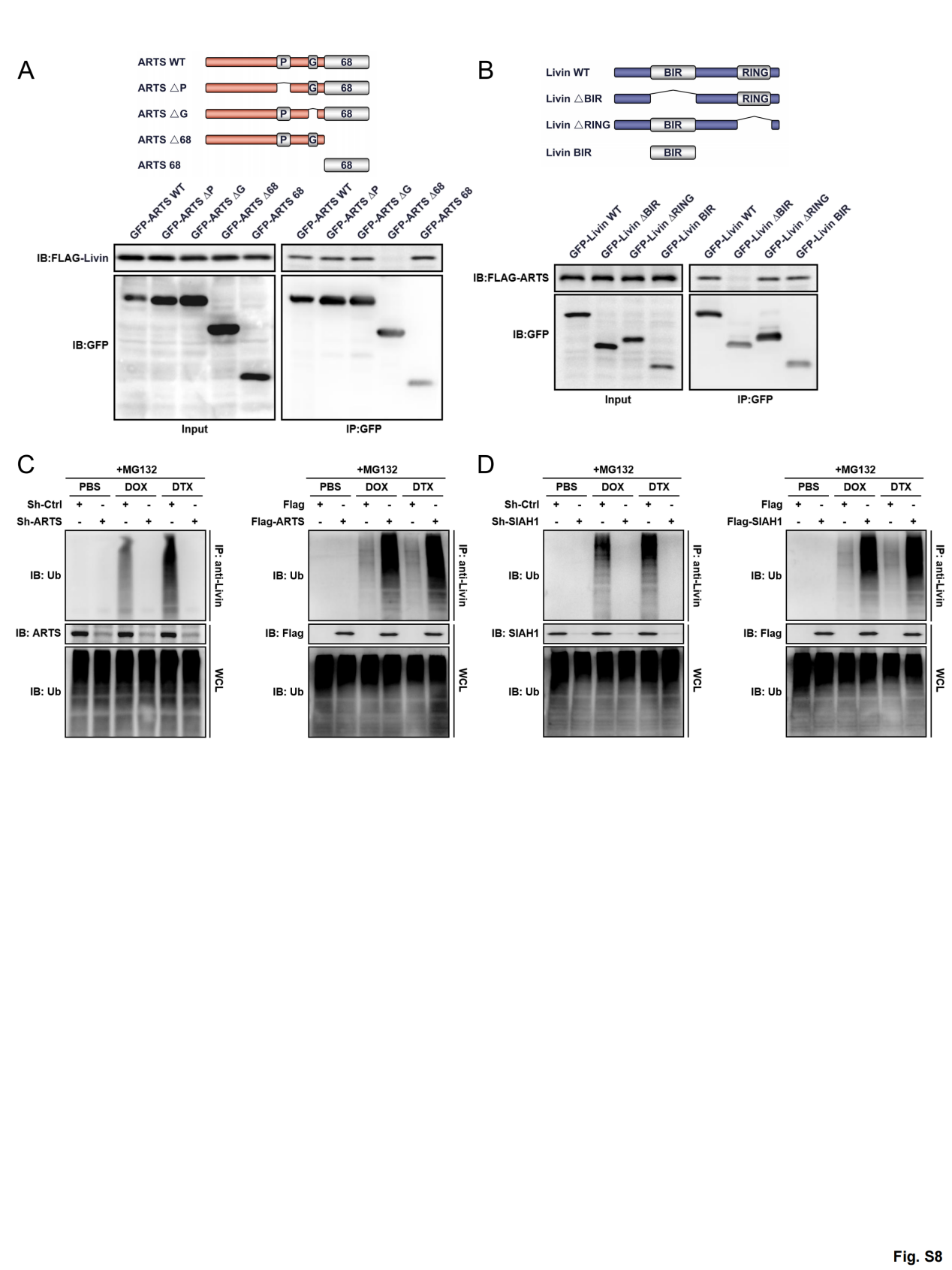


**Supplementary Figure 8. Structure-guided mapping of ARTS-Livin interfaces and ubiquitination assays. (A)** Domain schematics of ARTS constructs (WT; ΔP; ΔG; Δ68; 68-aa fragment) used for interaction mapping. HEK293T cells were co-transfected with GFP-tagged ARTS variants and FLAG-Livin. Lysates were subjected to anti-GFP immunoprecipitation (IP); both input and IP fractions were immunoblotted with anti-FLAG and anti-GFP to document expression and pulldown. **(B)** Domain schematics of Livin constructs (WT; ΔBIR; ΔRING; isolated BIR) used in reciprocal mapping. HEK293T cells were co-transfected with GFP-Livin variants and FLAG-ARTS; lysates underwent anti-GFP IP, followed by immunoblot detection for FLAG-ARTS and GFP. **(C)** Ubiquitination of Livin under chemotherapy: MDA-MB-231 (sh-Ctrl or sh-ARTS) (left) and MCF-7 (Flag or Flag-ARTS) (right) were treated with PBS, DOX, or DTX in the presence of MG132 (to stabilize ubiquitinated species). Lysates were split; one portion was subjected to anti-Livin IP and immunoblotted with anti-ubiquitin to detect Ub-Livin; the other portion was run as whole-cell lysate (WCL) and immunoblotted with anti-Ub to show total ubiquitin smear. **(D)** Ubiquitination of Livin under chemotherapy: MDA-MB-231 (sh-Ctrl or sh-SIAH1) (left) and MCF-7 (Flag or Flag-SIAH1) (right) were treated with PBS, DOX, or DTX plus MG132. Lysates were divided for anti-Livin IP followed by anti-ubiquitin immunoblot (Ub-Livin) and for WCL blotted with anti-Ub.


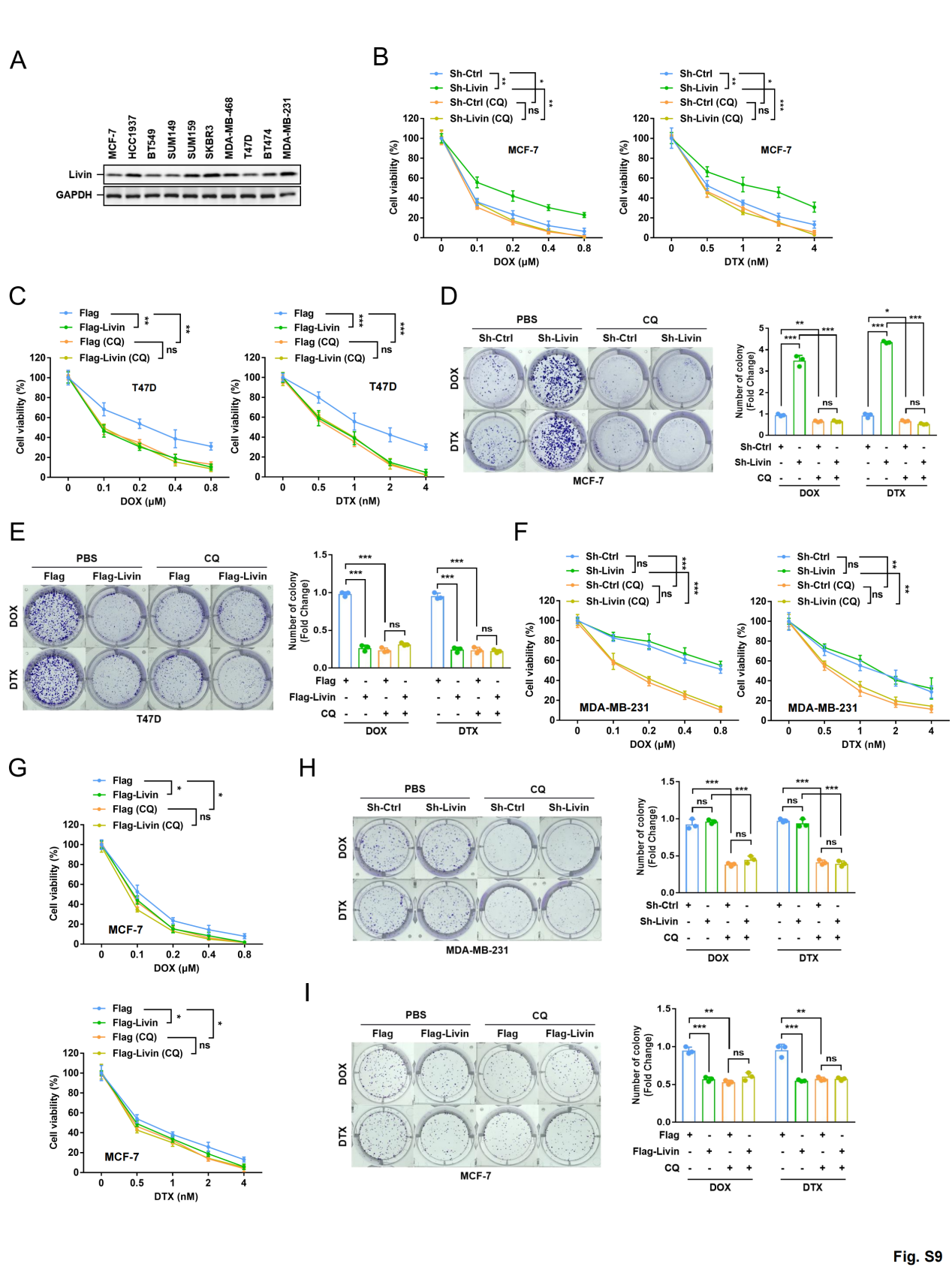


**Supplementary Figure 9. Livin mediates chemotherapy resistance through autophagy. (A)** Livin protein expression in breast cancer cell lines detected by immunoblot analysis. **(B-I)** Cells were treated as indicated and MTT and colony formation assays were performed.


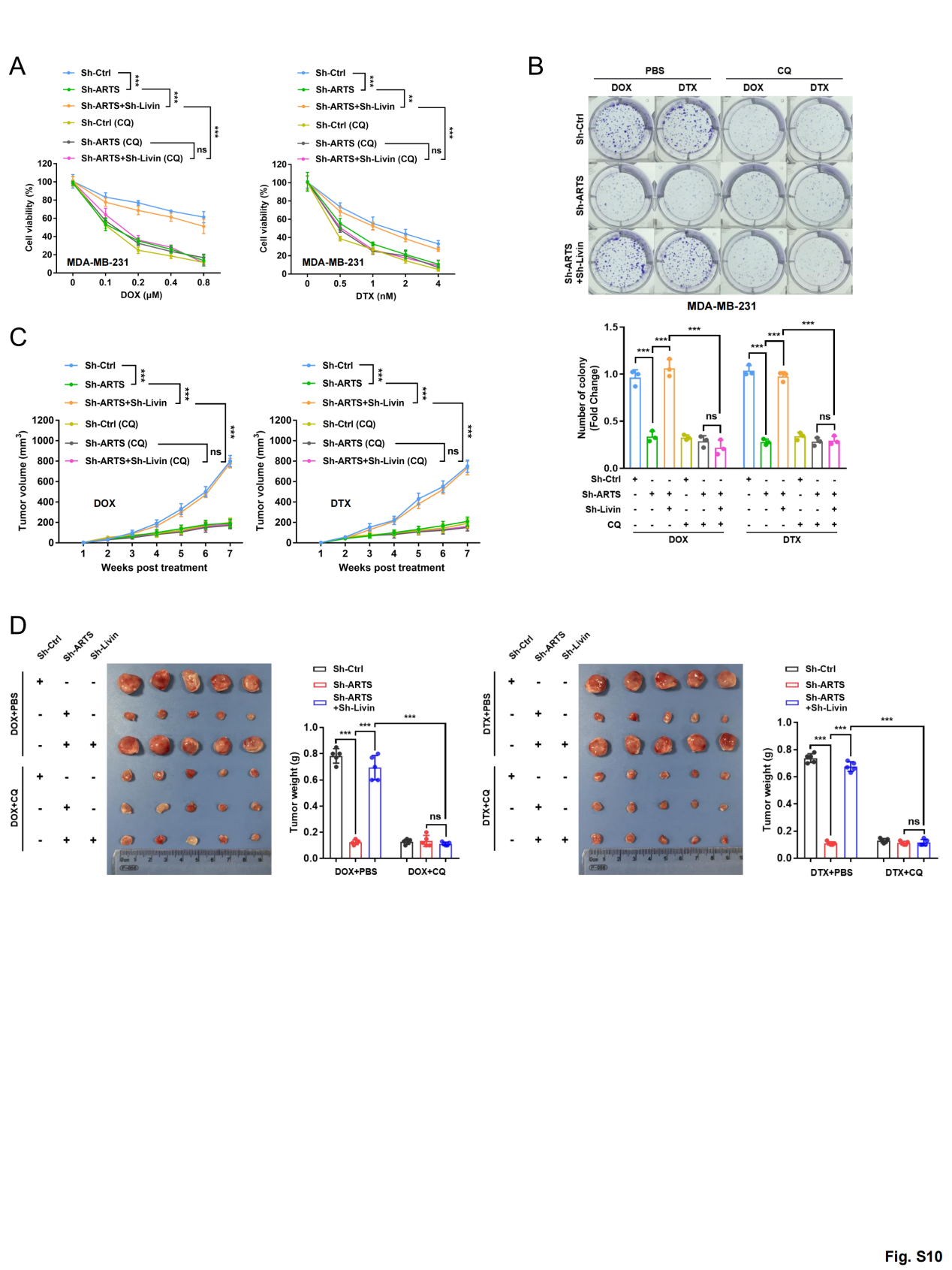


**Supplementary Figure 10. ARTS promotes apoptosis-dependent autophagy and chemoresistance via Livin. (A-B**) sh-Ctrl and sh-ARTS MDA-MB-231 cells transfected with sh-Livin were treated as indicated and (A) colony formation and (B) MTT assays were performed. **(C-D)** Sh-Ctrl and sh-ARTS MDA-MB-231 cells transfected with sh-Livin were injected into the second pair of breast fat pads of nude mice. DOX (2.5 mg/kg), DTX (5 mg/kg) or CQ (40 mg/kg) were administered as indicated twice a week for six consecutive weeks from the second week. Tumor growth curve, dissected tumor image and tumor weight of each group were individually analyzed and displayed as indicated (n = 5).


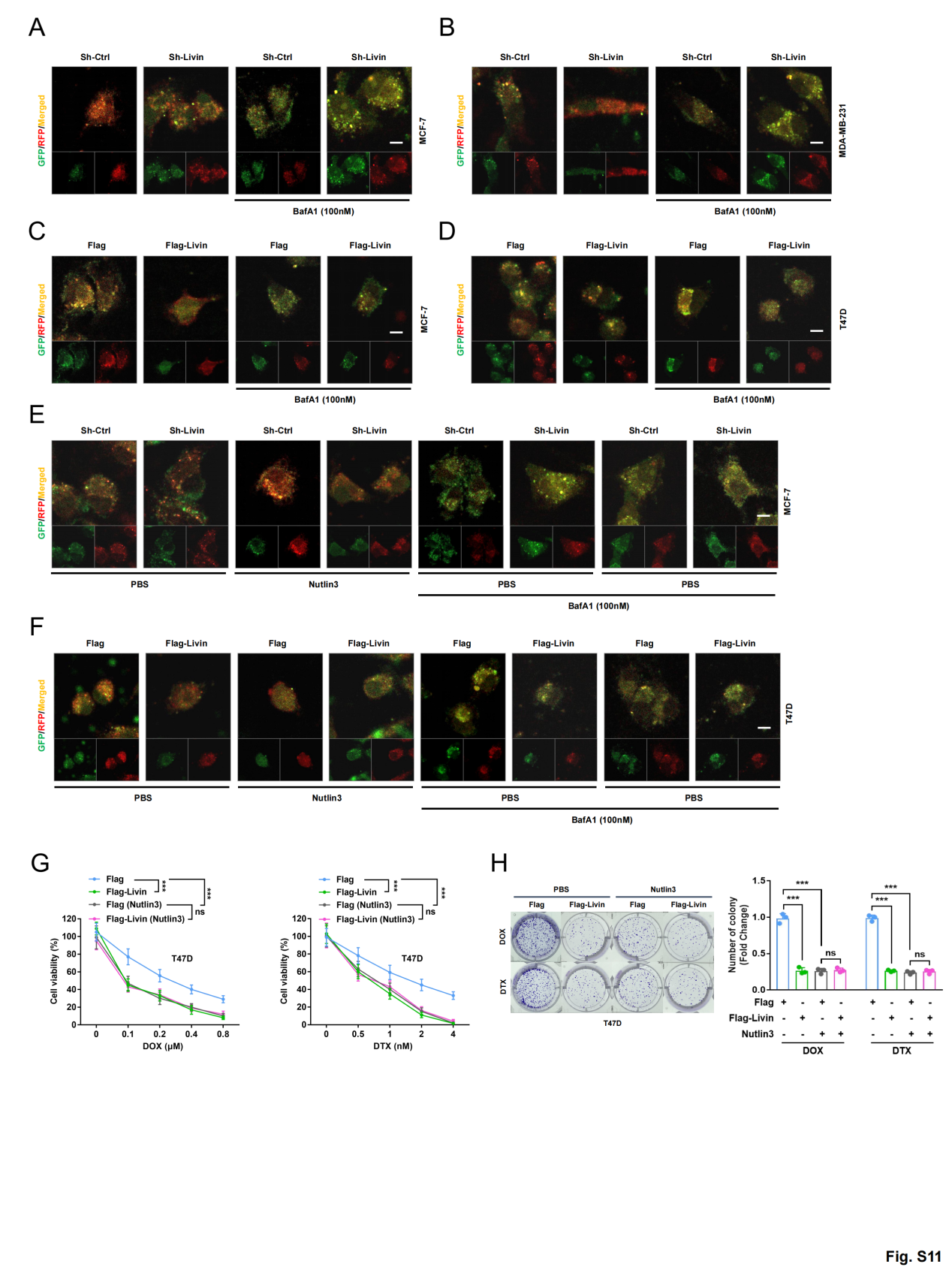


**Supplementary Figure 11. Tandem LC3 reporter imaging under Livin perturbation and Nutlin 3, with drug-response assays. (A-B)** Representative confocal images of mRFP-GFP-LC3 in MCF-7 and MDA-MB-231 stably expressing shCtrl or shLivin. Scale bar, 10 μm. **(C-D)** Representative images of mRFP-GFP-LC3 in MCF-7 and T47D expressing Flag or Flag-Livin. Scale bar, 10 μm. **(E-F)** Representative images of the same settings following Nutlin 3 treatment. Scale bar, 10 μm. **(G)** MTT viability of T47D cells (Flag or Flag-Livin) treated with graded DOX/DTX ± Nutlin 3. **(H)** Colony formation in T47D under the same genetic/drug conditions.


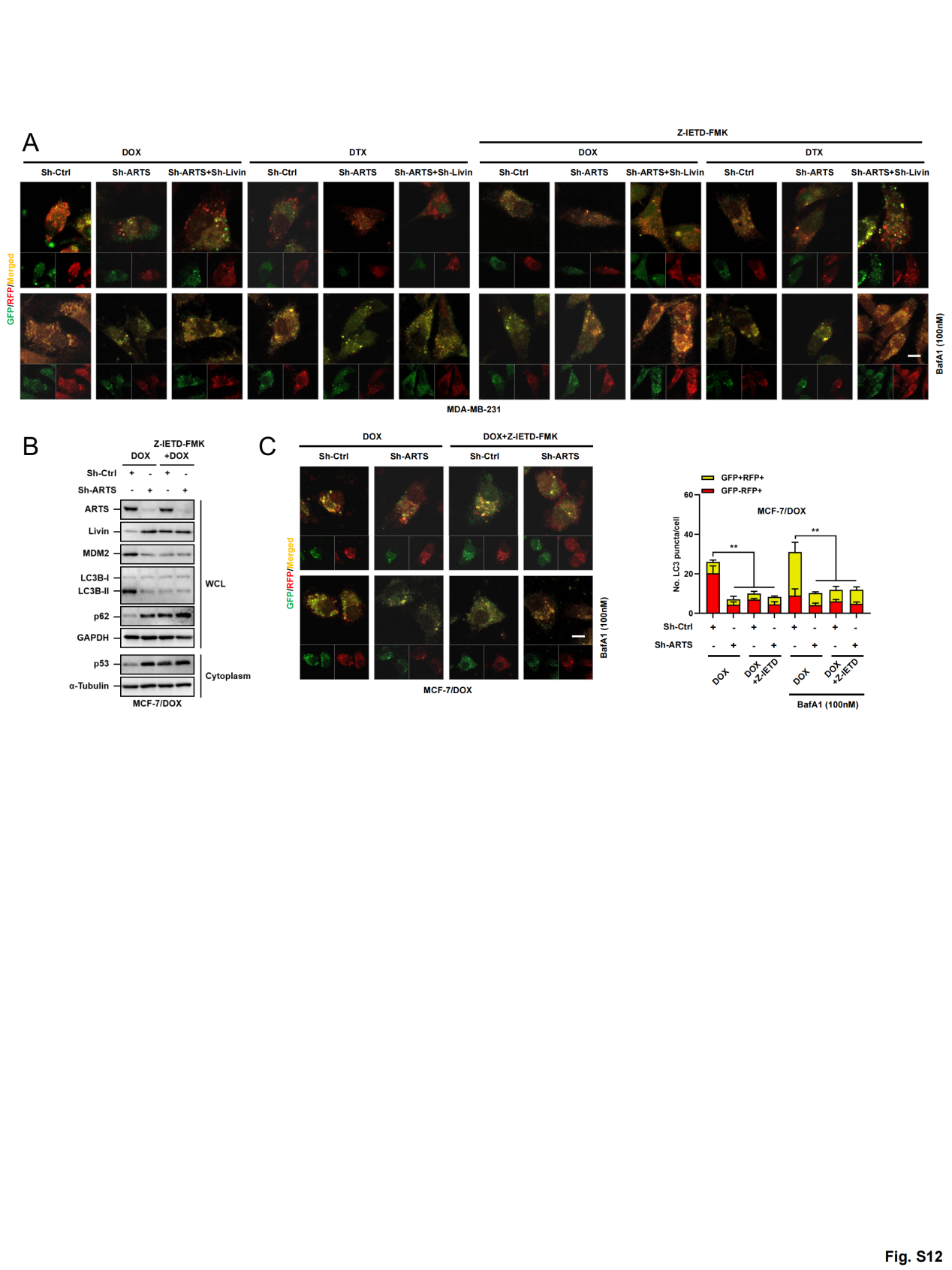
**Supplementary Figure 12. Caspase-8 blockade in LC3-flux imaging, with immunoblot profiling. (A)** Representative confocal images of tandem mRFP-GFP-LC3 in MDA-MB-231 cells stably expressing shCtrl, shARTS, or shARTS+shLivin. Cells were treated with DOX or DTX at the indicated. Scale bar, 10 μm. **(B)** MCF-7/DOX cells expressing shCtrl or shARTS were treated with DOX ± Z-IETD-FMK as indicated. Whole-cell lysates (WCL) were immunoblotted for ARTS, Livin, MDM2, LC3B-I/II, p62 with GAPDH loading control; cytosolic fractions were immunoblotted for p53 with α-tubulin as marker. **(C)** mRFP-GFP-LC3 in MCF-7/DOX cells (shCtrl or shARTS) under DOX ± Z-IETD-FMK, processed as in (A). Scale bar, 10 μm.


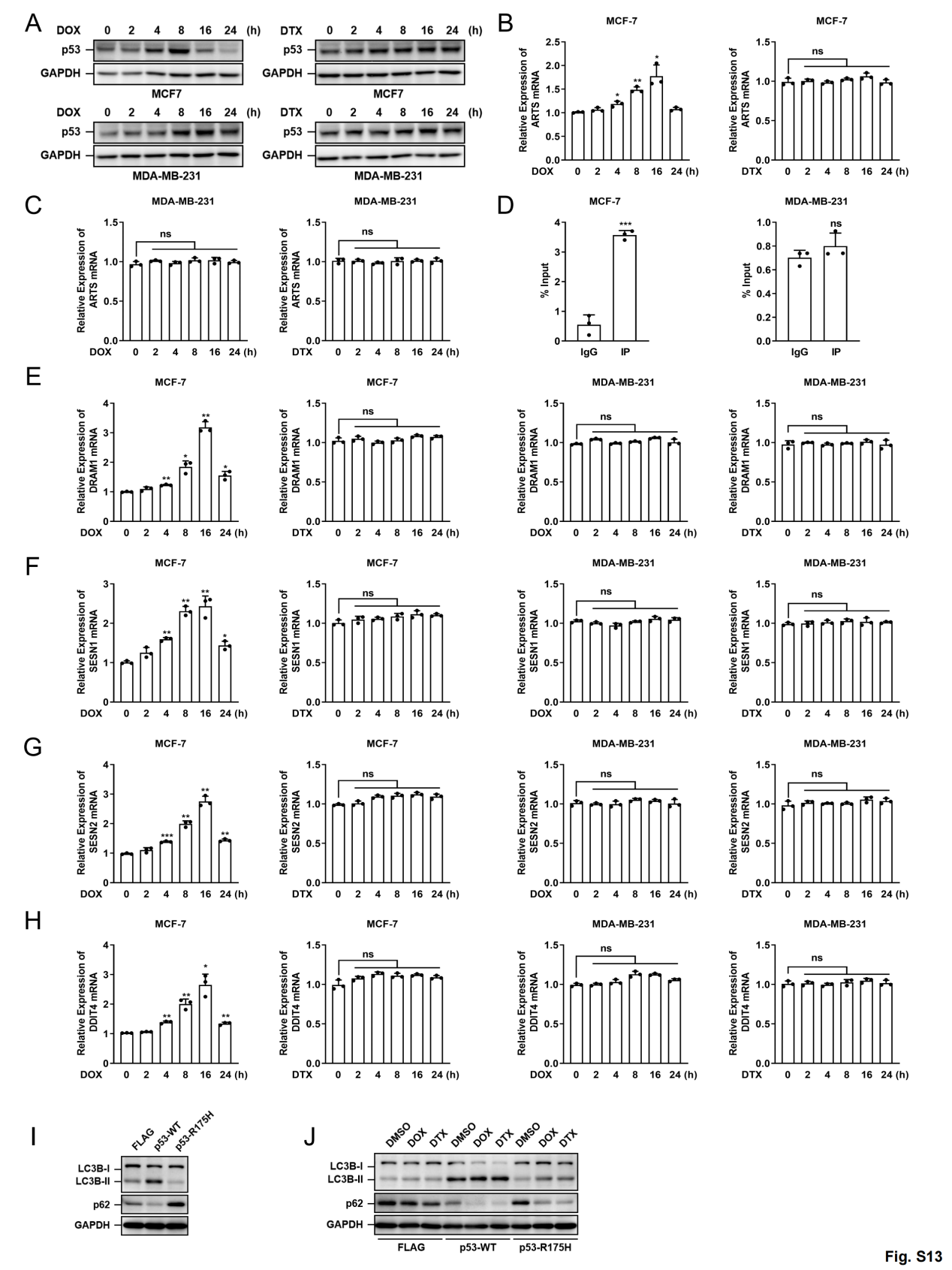
**Supplementary Figure 13. p53 dynamics with transcriptional readouts, ARTS promoter occupancy, and rescue in HCC1937. (A)** Time-course immunoblot of p53 in MCF-7 and MDA-MB-231 following treatment with DOX/DTX for the indicated hours; GAPDH served as loading control. **(B-C, E-H)** RT-qPCR time courses measuring ARTS (B) and canonical p53 target transcripts DRAM1 (E), SESN1 (F), SESN2 (G), and DDIT4 (H) after DOX or DTX in MCF-7 and MDA-MB-231. **(D)** ChIP-qPCR using anti-p53 or IgG in MCF-7 and MDA-MB-231; chromatin was cross-linked, sonicated (~200-500 bp), immunoprecipitated, and analyzed by qPCR targeting the ARTS promoter. **(I-J)** HCC1937 cells (p53-deficient) transfected with FLAG vector, p53-WT, or p53-R175H were processed for autophagy readouts.

**Supplementary Table 1. Association of ARTS expression with clinicopathological parameters of breast cancer in patients pre- and post-neoadjuvant chemotherapy**

| Patient | pre-NAC ARTS expression | | *P-*value | post-NAC ARTS expression | | *P-*value |
| --- | --- | --- | --- | --- | --- | --- |
|  | high, *n* | low, *n* |  | high, *n* | low, *n* |  |
| **Miller-Payne score** |  |  |  |  |  |  |
| 1-2 | 22 | 14 | **0.0007** | 24 | 12 | **0.0366** |
| 3-4 | 11 | 35 |  | 20 | 26 |  |
| **Ki-67** |  |  |  |  |  |  |
| ≤14% | 11 | 11 | 0.2753 | 12 | 19 | **0.0343** |
| >14% | 22 | 38 |  | 32 | 19 |  |
| **Age (years)** |  |  |  |  |  |  |
| ≤35 | 7 | 4 | 0.1079 | 6 | 5 | 0.9999 |
| >35 | 26 | 45 |  | 38 | 33 |  |
| **Lymph node metastasis** |  |  |  |  |  |  |
| No | 19 | 24 | 0.4447 | 22 | 21 | 0.6342 |
| Yes | 14 | 25 |  | 22 | 17 |  |
| **TNM stage** |  |  |  |  |  |  |
| I+II | 13 | 23 | 0.4996 | 19 | 17 | 0.8875 |
| III+IV | 20 | 26 |  | 25 | 21 |  |
| **ER** |  |  |  |  |  |  |
| Positive | 16 | 23 | 0.8907 | 20 | 19 | 0.6811 |
| Negative | 17 | 26 |  | 24 | 19 |  |
| **PR** |  |  |  |  |  |  |
| Positive | 18 | 23 | 0.4993 | 22 | 19 | 0.9999 |
| Negative | 15 | 26 |  | 22 | 19 |  |
| **HER-2** |  |  |  |  |  |  |
| Low | 28 | 34 | 0.1250 | 37 | 25 | 0.0543 |
| High | 5 | 15 |  | 7 | 13 |  |

ER: estrogen receptor; PR: progesterone receptor; HER-2: human epidermal growth factor receptor 2.

**Supplementary Table 2. Proteins that were present in both the DOX and DTX groups but absent in the PBS group**

| Protein name | AHCY, ALDH9A1, APOD, ASPRV1, ATP5F1A, BIRC2, BIRC3, CAPZB, CPA4, ERICH3, FGD6, IDE, IDH1, Livin, MYL6, NONO, NUDT5, PEBP1, PPIA, PPIB, RPL23, RPL26, RPL8, RPS4X, SEC61B, SIAH1, SOD1, SYNCRIP, TRIM21, TXN, VCL, YOD1, YWHAQ, TUBB4B/TUBB4A, IGKV2-29/IGKV2D-26, SPRR2B/SPRR2D, CAPZA1/CAPZA2, ATAD3C/ATAD3B, PLCL1/PLCL2, ACTN1/ACTN2 |
| --- | --- |

**Supplementary Table 3. Association of Livin expression with clinicopathological parameters of breast cancer in patients pre- and post-neoadjuvant chemotherapy**

| Patient | pre-NAC Livin expression | | *P-*value | post-NAC Livin expression | | *P-*value |
| --- | --- | --- | --- | --- | --- | --- |
|  | high, *n* | low, *n* |  | high, *n* | low, *n* |  |
| **Miller-Payne score** |  |  |  |  |  |  |
| 1-2 | 26 | 10 | 0.8638 | 12 | 24 | **0.0022** |
| 3-4 | 34 | 12 |  | 31 | 15 |  |
| **Ki-67** |  |  |  |  |  |  |
| ≤14% | 18 | 4 | 0.4013 | 22 | 9 | **0.0088** |
| >14% | 42 | 18 |  | 21 | 30 |  |
| **Age (years)** |  |  |  |  |  |  |
| ≤35 | 8 | 3 | 0.9999 | 5 | 6 | 0.7493 |
| >35 | 52 | 19 |  | 38 | 33 |  |
| **Lymph node metastasis** |  |  |  |  |  |  |
| No | 34 | 9 | 0.2055 | 22 | 21 | 0.8080 |
| Yes | 26 | 13 |  | 21 | 18 |  |
| **TNM stage** |  |  |  |  |  |  |
| I+II | 24 | 12 | 0.2396 | 19 | 17 | 0.9567 |
| III+IV | 36 | 10 |  | 24 | 22 |  |
| **ER** |  |  |  |  |  |  |
| Positive | 29 | 10 | 0.8171 | 23 | 16 | 0.2591 |
| Negative | 31 | 12 |  | 20 | 23 |  |
| **PR** |  |  |  |  |  |  |
| Positive | 32 | 9 | 0.3188 | 24 | 17 | 0.2689 |
| Negative | 28 | 13 |  | 19 | 22 |  |
| **HER-2** |  |  |  |  |  |  |
| Low | 43 | 19 | 0.2477 | 30 | 32 | 0.1958 |
| High | 17 | 3 |  | 13 | 7 |  |

ER: estrogen receptor; PR: progesterone receptor; HER-2: human epidermal growth factor receptor 2.

**Supplementary Table 4. Association of MDM2 expression with clinicopathological parameters of breast cancer in patients pre- and post-neoadjuvant chemotherapy**

| Patient | pre-NAC MDM2 expression | | *P-*value | post-NAC MDM2 expression | | *P-*value |
| --- | --- | --- | --- | --- | --- | --- |
|  | high, *n* | low, *n* |  | high, *n* | low, *n* |  |
| **Miller-Payne score** |  |  |  |  |  |  |
| 1-2 | 7 | 29 | 0.8114 | 20 | 16 | **0.0219** |
| 3-4 | 8 | 38 |  | 14 | 32 |  |
| **Ki-67** |  |  |  |  |  |  |
| ≤14% | 6 | 16 | 0.2028 | 9 | 22 | 0.0748 |
| >14% | 9 | 51 |  | 25 | 26 |  |
| **Age (years)** |  |  |  |  |  |  |
| ≤35 | 1 | 10 | 0.6794 | 6 | 5 | 0.5123 |
| >35 | 14 | 57 |  | 28 | 43 |  |
| **Lymph node metastasis** |  |  |  |  |  |  |
| No | 10 | 33 | 0.2631 | 17 | 26 | 0.7097 |
| Yes | 5 | 34 |  | 17 | 22 |  |
| **TNM stage** |  |  |  |  |  |  |
| I+II | 9 | 27 | 0.1646 | 14 | 22 | 0.6755 |
| III+IV | 6 | 40 |  | 20 | 26 |  |
| **ER** |  |  |  |  |  |  |
| Positive | 7 | 32 | 0.9388 | 13 | 26 | 0.1547 |
| Negative | 8 | 35 |  | 21 | 22 |  |
| **PR** |  |  |  |  |  |  |
| Positive | 6 | 35 | 0.3915 | 14 | 27 | 0.1786 |
| Negative | 9 | 32 |  | 20 | 21 |  |
| **HER-2** |  |  |  |  |  |  |
| Low | 11 | 51 | 0.9999 | 27 | 35 | 0.4998 |
| High | 4 | 16 |  | 7 | 13 |  |

ER: estrogen receptor; PR: progesterone receptor; HER-2: human epidermal growth factor receptor 2.

**Supplementary Table 5: Antibodies**

| Antibody | Company | Catalog # | Species | Application |
| --- | --- | --- | --- | --- |
| ARTS | Sigma-Aldrich | SAB3500314 | Rabbit | WB: 1:2,000  IHC: 1:200  IF: 1:500 |
| ARTS | Sigma-Aldrich | A4471 | Mouse | IP: 1 μg  IF: 1:500 |
| LC3B | Sigma-Aldrich | L7543 | Rabbit | WB: 1:2,000 |
| IgG | Proteintech | B900610 | Rabbit | IP: 1 μg |
| Livin | Proteintech | 27543-1-AP | Rabbit | IP: 1 μg  WB: 1:2,000  IHC: 1:500  IF: 1:500 |
| GFP | Proteintech | 50430-2-AP | Rabbit | IP: 1 μg  WB: 1:2,000 |
| Flag | Proteintech | 66008-4-Ig | Mouse | WB: 1:5,000 |
| VDAC1 | SANTA CRUZ | sc-390996 | Mouse | WB: 1:1,000 |
| α-Tubulin | SANTA CRUZ | sc-5286 | Mouse | WB: 1:1,000 |
| Lamin B1 | SANTA CRUZ | sc-374015 | Mouse | WB: 1:1,000 |
| Ubiquitin | SANTA CRUZ | sc-8017 | Mouse | WB: 1:1,000 |
| SIAH1 | Origene | TA365015 | Rabbit | WB: 1:2,000 |
| MDM2 | Proteintech | 66511-1-Ig | Mouse | IP: 1 μg  WB: 1:2,000  IHC: 1:500 |
| p53 | Origene | TA502870 | Mouse | WB: 1:2,000  IHC: 1:500 |
| GAPDH | Origene | TA802519 | Mouse | WB: 1:2,000 |
| TOM20 | Proteintech | 11802-1-AP | Rabbit | IF: 1:500 |
| c-Caspase 3 | Proteintech | 25128-1-AP | Rabbit | WB: 1:1,000 |
| c-PARP1 | Abcam | ab32561 | Rabbit | WB: 1:1,000 |

**Supplementary** **Table 6:** **Agents**

| Agent | Company | Catalog # |
| --- | --- | --- |
| BI-6C9 | MedChemExpress | HY-103661 |
| Bz-423 | MedChemExpress | HY-13108 |
| Chloroquine | ALADDIN | C193834 |
| CHX | MedChemExpress | HY-12320 |
| Docetaxel | ALADDIN | D107320 |
| Doxorubicin | ALADDIN | A183027 |
| MG132 | ALADDIN | M126521 |
| MSN-125 | MedChemExpress | HY-120079 |
| Nutlin3 | ALADDIN | N129969 |
| Z-DEVD-FMK | MedChemExpress | HY-12466 |
| Z-IETD-FMK | MedChemExpress | HY-101297 |
| Z-LEHD-FMK | MedChemExpress | HY-P1010 |
| Z-VAD-FMK | ALADDIN | Z408507 |
| Z-YVAD-FMK | MedChemExpress | HY-P1009 |
| Bafilomycin A1 | MedChemExpress | HY-100558 |
| Rapamycin | MedChemExpress | HY-10219 |
| Earle’s Balanced Salt Solution | Beyotime | C0213 |

**Supplementary Table 7: Primers and oligonucleotides sequences used in this study**

| **Oligonucleotide sequence of shRNAs** | |
| --- | --- |
| ARTS shRNA1 | 5'-CCGGGCCTGAGGCTCCTGGCACCCTCGAGGGTGCCAGGAGCCTCAGGCTTTTTG-3' |
|  | 5'-AATTCAAAAAGCCTGAGGCTCCTGGCACCCTCGAGGGTGCCAGGAGCCTCAGGC-3' |
| ARTS shRNA2 | 5'-CCGGCAGGCCAAGAGCACCAGGGCTCGAGCCCTGGTGCTCTTGGCCTGTTTTTG-3' |
|  | 5'-AATTCAAAAACAGGCCAAGAGCACCAGGGCTCGAGCCCTGGTGCTCTTGGCCTG-3' |
| ARTS shRNA3 | 5'-CCGGGAGCACCAGGGGCAGGGCTCTCGAGAGCCCTGCCCCTGGTGCTCTTTTTG-3' |
|  | 5'-AATTCAAAAAGAGCACCAGGGGCAGGGCTCTCGAGAGCCCTGCCCCTGGTGCTC-3' |
| SIAH1 shRNA | 5'-CCGGTCACCAGCAGTTCTTCGCAATCTCGAGATTGCGAAGAACTGCTGGTGATTTTTG-3' |
|  | 5'-AATTCAAAAATCACCAGCAGTTCTTCGCAATCTCGAGATTGCGAAGAACTGCTGGTGA-3' |
| Livin shRNA2 | 5'-CCGGCGCCGTGTCCATCGTCTTTGTCTCGAGACAAAGACGATGGACACGGCGTTTTTG-3' |
|  | 5'-AATTCAAAAACGCCGTGTCCATCGTCTTTGTCTCGAGACAAAGACGATGGACACGGCG-3' |
| ATG7 shRNA | 5'-CCGGGCTTTGGGATTTGACACATTTCTCGAGAAATGTGTCAAATCCCAAAGCTTTTTG-3' |
|  | 5'-AATTCAAAAAGCTTTGGGATTTGACACATTTCTCGAGAAATGTGTCAAATCCCAAAGC-3' |
| Casp-8 shRNA | 5'-CCGGGACATGAACCTGCTGGATATTCTCGAGAATATCCAGCAGGTTCATGTCTTTTT-3' |
|  |  |
| **Primers for cloning** | |
| ARTS-CDS | 5'-ATATACGCGTATGATCAAGCGTTTCCTGGA-3' |
|  | 5'-ATATACTAGTCTAGTGGCAGCCCTGCCCCT-3' |
| Livin-CDS | 5'-ATATACGCGTATGGGACCTAAAGACAGTGC-3' |
|  | 5'-ATATACTAGTCTAGGACAGGAAGGTGCGCA-3' |
| SIAH1-CDS | 5'-ATATACGCGTATGAGCCGTCAGACTGCTAC-3' |
|  | 5'-ATATACTAGTTCAACACATGGAAATAGTTA-3' |
| ATG7-CDS | 5'-ATATACGCGTATGGCGGCAGCTACGGGGGA-3' |
|  | 5'-ATATACTAGTTCAGATGGTCTCATCATCGC-3' |
| **Primers for qPCR** | |
| ARTS | 5'-CCGAAAGTCCGTGAAGAAA-3' |
|  | 5'-TGTCCACAATGGTGAGCC-3' |
| DRAM1 | 5'-TGGATTGGTGGGATGTT-3' |
|  | 5'-AGATGATGGACTGTAGGAGC-3' |
| SESN1 | 5'-GCTTTCAAATACCGAGTC-3' |
|  | 5'-CCAAGTTCCTGGATGCT-3' |
| SESN2 | 5'-CTGCACCCTGACTACTTTACCA-3' |
|  | 5'-GCCACAGCCAAACACGAA-3' |
| DDIT4 | 5'-TCGTCCACCTCCTCTTCGC-3' |
|  | 5'-AGCCACTGTTGCTGCTGTCC-3' |
| **Primers for ChIP-qPCR** | |
| ARTS | 5’-GTATTAGACCCTGCCTCCATCA-3’ |
|  | 5’-GAAGACTGACTTTGAGCCATCC-3’ |
